# Krypton and the Fundamental Flaw of the Lennard-Jones Potential

**DOI:** 10.1021/acs.jpclett.2c02004

**Published:** 2022-08-29

**Authors:** Ciprian G. Pruteanu, John S. Loveday, Graeme J. Ackland, John E. Proctor

**Affiliations:** † SUPA, School of Physics and Astronomy and Centre for Science at Extreme Conditions, The University of Edinburgh, Edinburgh EH9 3FD, U.K.; ‡ Materials & Physics Research Group, Newton Building, University of Salford, Manchester M5 4WT, U.K.

## Abstract

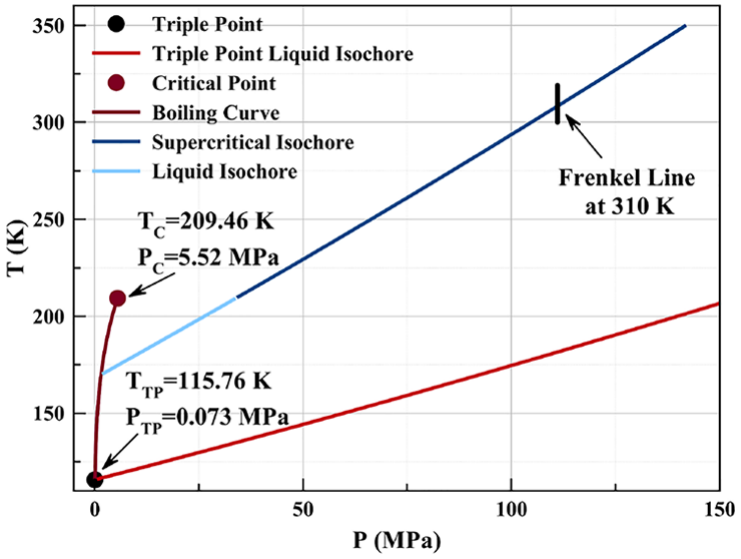

We have performed a series of neutron scattering experiments on supercritical krypton. Our data and analysis allow us to characterize the Frenkel line crossover in this model monatomic fluid. The data from our measurements was analyzed using Empirical Potential Structure Refinement to determine the short- and medium-range structure of the fluids. We find evidence for several shells of neighbors which form approximately concentric rings of density about each atom. The ratio of second to first shell radius is significantly larger than in any crystal structure. Modeling krypton using a Lennard-Jones potential is shown to give significant errors, notably that the liquid is overstructured. The true potential appears to be longer ranged and with a softer core than the 6–12 powerlaws permit.

Neutron diffraction is a powerful technique for the study of the structure of noncrystalline materials. The key to obtaining accurate structural information in such systems is the ability to access as wide a range of momentum transfers (*Q*) as possible.^1^ Neutrons scatter from the nucleus, which on the atomic scale is a point scatterer, and so neutrons are not subjected to the form-factor falloff which limits the maximum *Q* that can be accessed by X-rays. In addition, neutron cross sections vary much less than for X-rays and vary pseudorandomly with atomic mass.^1^ As a result, in multiatom systems, there is a tendency for all species to contribute more equally to the diffraction pattern. This is particularly true for light elements like hydrogen, oxygen and nitrogen all of which have relatively large neutron cross sections.^1^ In addition, the pseudo random variation means that different isotopes of the same element scatter neutrons very differently. This can be exploited by replacing one isotope with another to measure directly the partial scattering factors in multiatom systems, a technique known as isotopic substitution. Neutron diffraction also has other advantages which are relevant to noncrystalline systems. Attenuation is generally weaker than for X-rays and more readily calculable and hence correctable.^1^ As a result of these advantages, neutron diffraction is generally the technique of choice for the study of liquids and amorphous systems and many thousands of such systems have been studied.

An area where neutron studies have just started is the determination of the Frenkel line. This is a line on the phase diagram which separates the supercritical fluid into regions between typical fluid behavior and dynamical properties more often associated with solids, such as a fully coordinated neighbor shell and the propagation of high frequency shear waves.

While the Frenkel line has been identified using Raman spectroscopy^2−4^ and (in one case) with X-ray diffraction,^5^ neutron diffraction has key advantages for studying noncrystalline systems as outlined above. The Frenkel line has been identified using neutron diffraction both by ourselves^4,6^ and by other authors.^7^ However, disagreement remains as to what actually changes when the Frenkel line is crossed.

The neutron studies to date have focused on molecular systems like N_2_ and CO_2_. Here, we instead focus on the archetypal monatomic system of Kr. This enables us to conduct a study in which fewer approximations are made in the analysis, and—crucially—to extend the scope of the study to look at the changes in the “effective” interatomic potential that take place when the Frenkel line is crossed.

In general, the energy of a system can be defined by a Hamiltonian , a function of the set of atomic positions {**r**}. Using  the partition function can be sampled by a standard Monte Carlo process, and thereby all structural and thermodynamic properties determined. Unfortunately,  is not known.

However, neutron scattering measures *S*(*q*) . There is no way to uniquely determine a general many-variable function  from the single-variable function *S*(*q*). “Empirical Potential Structure Refinement” (EPSR) postulates that  can be approximated as a sum of pairwise potentials *V*(*r*).

This raises several issues:

- Can *V*(*r*) be uniquely determined from *S*(*q*) in principle?
- Is the experimental data good enough to do this determination in practice?
- Can we test how good an approximation to  is *V*(*r*)?
- Does *V*(*r*) have a strong density or temperature dependence?
- Is there a more parsimonious way to fit the data, and if so, does it have physical meaning?

The EPSR method simultaneously and self-consistently refines the structure and the interatomic pairwise potential *V*(*r*) to fit the measured *S*(*q*) .

EPSR refinement defines *V*(*r*) in terms of some parameters, some of which are assumed *a priori*—typically a physically motivated force-field fitted to the material at hand, some of which are adjustable. It then runs a Monte Carlo process on the positions of a set of simulated molecules in a periodic box to generate a model, *S*_*calc*_(*q*). It then uses another Monte Carlo process on the adjustable potential parameters to minimize the difference between *S*(*q*) and *S*_*calc*_(*q*). These refinements are continued until a self-consistent solution is found: a *V*(*r*) which gives the best fit to the observed experimental *S*(*q*) .

EPSR requires a starting guess for the potential *V*(*r*), for krypton the Lennard-Jones potential is a typical choice. The inert gases are generally regarded as the simplest elemental systems, interacting only via van der Waals interactions. The van der Waals interaction, also called London dispersion, arises from induced dipole–dipole interactions. These fall off with atomic separation as *r*^–6^. This physics is encapsulated in the Lennard-Jones potential:
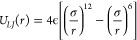
where *r* is the distance between two particles, ϵ is the depth of the potential (“dispersion energy”), and σ is the distance at which the potential energy is 0 (size of the particle). The minimum of the potential occurs at *r* = 2^1/6^σ, representing the equilibrium separation of a dimer. In a condensed phase, the near-neighbor separation is typically shorter.

The repulsive component, the (σ/*r*)^12^ term, represents the short-ranged screened Coulomb nuclear repulsion and the Pauli exclusion. The twelfth power is mainly chosen for ease of computation, being the square of the attractive term, and is not connected to any physical theory. The Lennard-Jones potential also contains the assumption that the interactions are pairwise additive. In the induced dipole picture, this implies that an atom can correlate its fluctuations simultaneously with all its neighbors, just as effectively as it could in a dimer. However, the attraction comes from antiferroelectric correlations. So frustration in a condensed phase will lead to weaker bonds. In the quantum picture, the same physics plays out through second order perturbation theory.

In this paper, we report neutron scattering experiments and refinements on fluid krypton. We use the measured *S*(*q*) and performed EPSR in order to determine the liquid structure. The EPSR process tests how good a description of the data the Lennard-Jones potentials give. The main feature expected in the data is the Frenkel line.

## Methods

*Experimental Procedure.* Neutron scattering was performed on the SANDALS diffraction instrument at the ISIS Pulsed Neutron Source based at Rutherford-Appleton Laboratory (RAL), Oxfordshire, UK. Samples of pure Kr (research grade N5, 99.999% Kr) were loaded in a TiZr can, and the pressure was monitored using a pressure intensifier fitted to the cell. The density was determined from the appropriate pressures using the Kr equation of state by Lemmon and Span.^8^ The temperature was maintained constant at 310 K for all pressures. Diffraction patterns were collected for ca. 12 h for each pressure point, varied according to sample density.

*Empirical Potential Structure Refinement.* As stated above, EPSR is a Monte Carlo-based method for the extraction of structural information from total scattering data obtained from disordered systems. A simulation box containing 5000 Kr atoms was fitted to every collected diffraction pattern. The Kr Lennard-Jones potential was defined using the parameters from Rutkai et al.^9^ (ϵ/*k*_*B*_ = 162.58 K, σ = 3.6274 Å), obtained by fitting to experimental equations of state for vapor pressure and saturated liquid density and adjusted so they correctly reproduce the experimental critical temperature (*T*_*C*_) and density. Once a sufficiently good agreement to the data was obtained, the empirical potential refinement was stopped, and 5000+ configurations were accumulated in order to sample adequately the pair distribution functions (PDFs).

*Criteria for the Identification of the Frenkel Line.* In recent years, it has been shown that the Frenkel line constitutes a crossover in the dynamical properties and behavior of a given system.^10,11^ In addition, there are structural markers accompanying this crossover that are readily detectable in diffraction measurements. The most evident is a plateauing of the coordination number with increasing pressure, as noted by Prescher et al.^5^ in Ne and by Proctor, Pruteanu et al. in N_2_.^4^ Previous work on nitrogen has also shown the effect of temperature of the Frenkel line and distinguished it from the Widom lines.^12^ With the aid of a machine-learned classical force-field for molecular nitrogen, an analytical expression (Pruteanu–Ackland equation) for the location of the Frenkel line in N_2_ was proposed:^6^
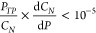
1Here *P*_*TP*_ is the triple point pressure, *P* is the pressure of the system, and *C*_*N*_ is the coordination number defined as the integral of the pair distribution function up to the distance of the first nonzero minimum. The same criterion involving the variation of the log of the coordination number as a function of pressure is used in the current paper to identify the Frenkel line in krypton at 310 K.

**Figure 1:**
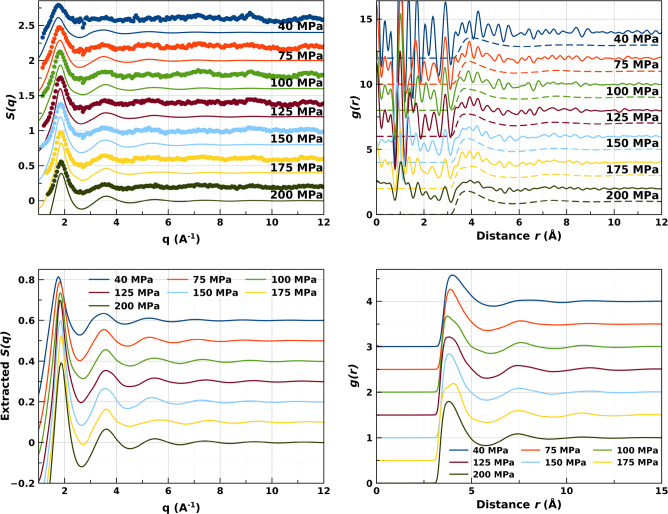
(Left) All EPSR fitted *S*(*q*)’s (continuous lines) and collected data (points). (Right) Pair Distribution Functions of krypton, both raw transformed (continuous noisy line) and extracted using EPSR (dashed/continuous smooth line) at all pressures investigated in the current study. The onset of medium-range order (third “bump” appearing the PDF) is visible at pressure above 100 MPa in the simulation-extracted functions.

*The Frenkel Line in Krypton at 310 K.* The measured *S*(*q*)’s along with their raw transformed *G*(*r*) and the EPSR fits are presented below, in Figure 1. While all the fits yielded very good *R*-factors and quality factors (around 0.0005), we noticed a significant improvement in the signal-to-noise ratio, and hence the quality of the diffraction patterns as pressure/density was increased. This in itself is not surprising, as by increasing the density one increases the amount of material present in the beam and hence the diffracted signal, but it is worth mentioning as a fact to be aware of when performing similar measurement on fluid krypton. The full detailed analysis of the individual pressure points considered in this study can be found in the Supporting Information.

**Figure 2:**
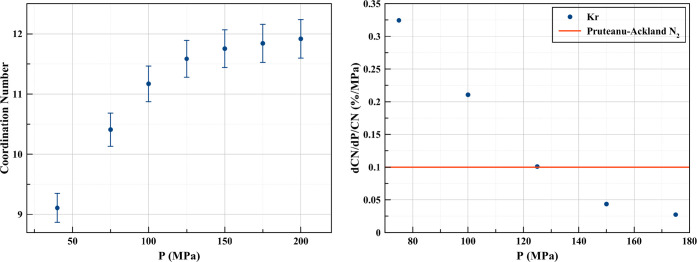
(Left) Coordination numbers of krypton at 310 K obtained from EPSR *g*(*r*)’s. A tendency to level off above 100 MPa is easily visible. (Right) Percentage change in coordination number of Kr as a function of pressure.

The exact application of the Pruteanu–Ackland criterion as formulated for nitrogen^6^ would indicate a different position for the Frenkel line in Kr than the one identified below. We do note however that the line in Kr is present at a similar log change of the coordination number with pressure (Figure. 2). From the measurements reported herein we conclude that for krypton at 310 K the Frenkel line is crossed at a pressure of ∼110 MPa.

Similar to previous measurements,^4,12^ the ratio between first and second shell radius is close to : the ratio of peak maxima in *g*(*r*) is 0.516(2) with no discernible pressure dependence. This can be contrasted with 0.707 for a close-packed crystal or 0.866 for bcc. The smaller ratios suggests that the structural three-dimensional motifs which allow close second neighbor approach (e.g., bipyramids) are not significantly present in the fluid: it appears that the RDF is more representative of a one-dimensional order, in density only. At pressures above the Frenkel line (>110 MPa), a third shell becomes increasingly visible in the pair distribution function, at about three times the radius of the first shell, suggesting the onset of medium-range order in the fluid’s structure.

The current measurements are consistent with those of Teitsma^13^ for low pressure krypton, where the highest pressure reached was 20 MPa (half the lowest pressure considered in the present study) and the accessible *q*-range was limited to 0.3–4 Å^–1^. The analysis of a few selected data sets from Teitsma using the same methods employed for our current measurements and their associated coordination numbers are presented in the Supporting Information. They show a smooth variation of the coordination number as a function of pressure throughout the entire extended pressure range, similar to that reported in Pruteanu et al.^6^

*Potential of Mean Force.* For simple fluids such as krypton, we can define the so-called potential of mean force from the pair distribution functions.^14,15^
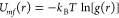
2The potential of mean force incorporates entropic effects such as the “depletion force” so, at best, it should be regarded as a Landau free energy. Terms beyond pairwise interactions become convolved in *U*_*mf*_ in an averaged way, the averaging depending on the density. Specifically, we note that applying the potential of mean force directly in a Monte Carlo or molecular dynamics calculation does not reproduce *g*(*r*). Even if *V*(*r*) were a hard-sphere potential, it would produce a *g*(*r*) and *U*_*mf*_(*r*) with long-range oscillatory structure.

**Figure 3:**
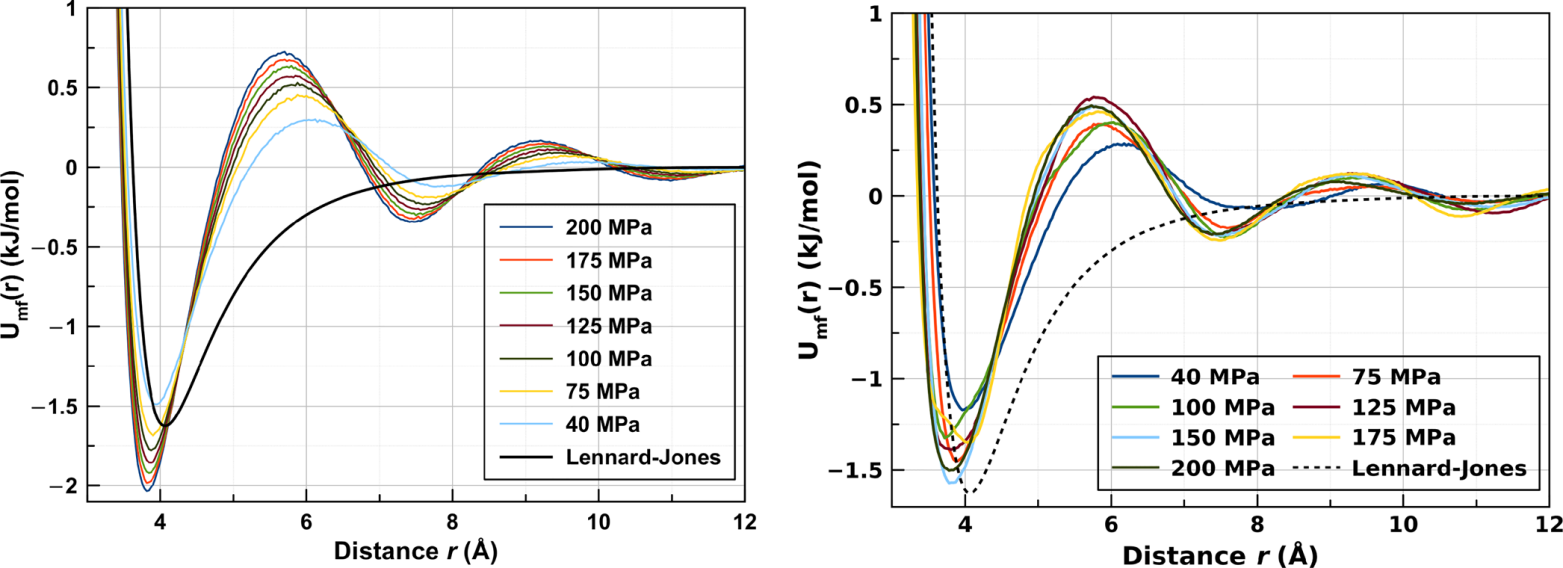
(Left) Potentials of mean force from pure Monte Carlo simulations using the Lennard-Jones potential derived according to *g*(*r*) from eq 2 and the Lennard-Jones potential itself. (Right) Potentials of mean force from fully fitted EPSR refinements derived according to *g*(*r*) from eq 2 for all the pressures considered in the present study.

We can see in Figure 3a that the potential of mean force deduced for a Lennard-Jones fluid exhibits several minima and becomes stronger than the potential itself. By contrast, the potential of mean force derived from the experimental data are weaker than Lennard-Jones. These *g*(*r*)’s are found using EPSR to eliminate noise in *g*(*r*) from the direct Fourier transform of the experimental *S*(*q*) . Thus, we can already conclude from *g*(*r*) that the Lennard-Jones model potential will overstructure the liquid (full LJ *g*(*r*)’s and coordination numbers are presented in parts S16 and S17 in the Supporting Information).

*Density Variation of Effective Potential.* For a given interatomic potential *V*(*r*) the associated radial distribution function *g*(*r*) is unique and can be determined by molecular dynamics or Monte Carlo simulation. In the special case of pair-potentials, the inverse is also true: *g*(*r*) uniquely specifies *V*(*r*).^16^ Even with a many-body potential *g*(*r*) from liquid data can reparameterise the exact interatomic potential which created it,^17^ provided the functional form is known. However, as noted initially by Soper^18^ and restated recently by Zhao,^19^ EPSR is unable to determine quantitatively the true form of the potential for a given system. Nevertheless, it may be possible to use it to determine shortcomings in the model potentials used to describe the atoms (such as Lennard-Jones models). Below, we show the effective potentials (the sum between the empirical potential and the Lennard-Jones model) used to adequately fit our data at all pressures, and contrast them to a pure Lennard-Jones potential.

For all the data sets measured, the empirical potential always acts so as to soften the repulsive component of the Lennard-Jones potential. This implies that the repulsive part of the Lennard-Jones potential, described by the *r*^–12^ dependence and occasionally rationalized as accounting for Pauli exclusion effects, is too strong even in krypton, a system as simple and close to what Lennard-Jones aims to describe.

A similar situation is present for the attractive component as well, the *r*^–6^ functional form rationalized as London dispersion, increasing too abruptly at all pressures considered. This suggests that even as the density is increased, and hence the repulsive components becoming more present, the Lennard-Jones model is still not attractive enough to correctly represent krypton. For the lower pressure points, there is a significant and readily noticeable difference between Lennard-Jones-shaped potentials and the effective potential obtained from EPSR, with the latter showing markedly more attraction between Kr atoms than the former.

**Figure 4:**
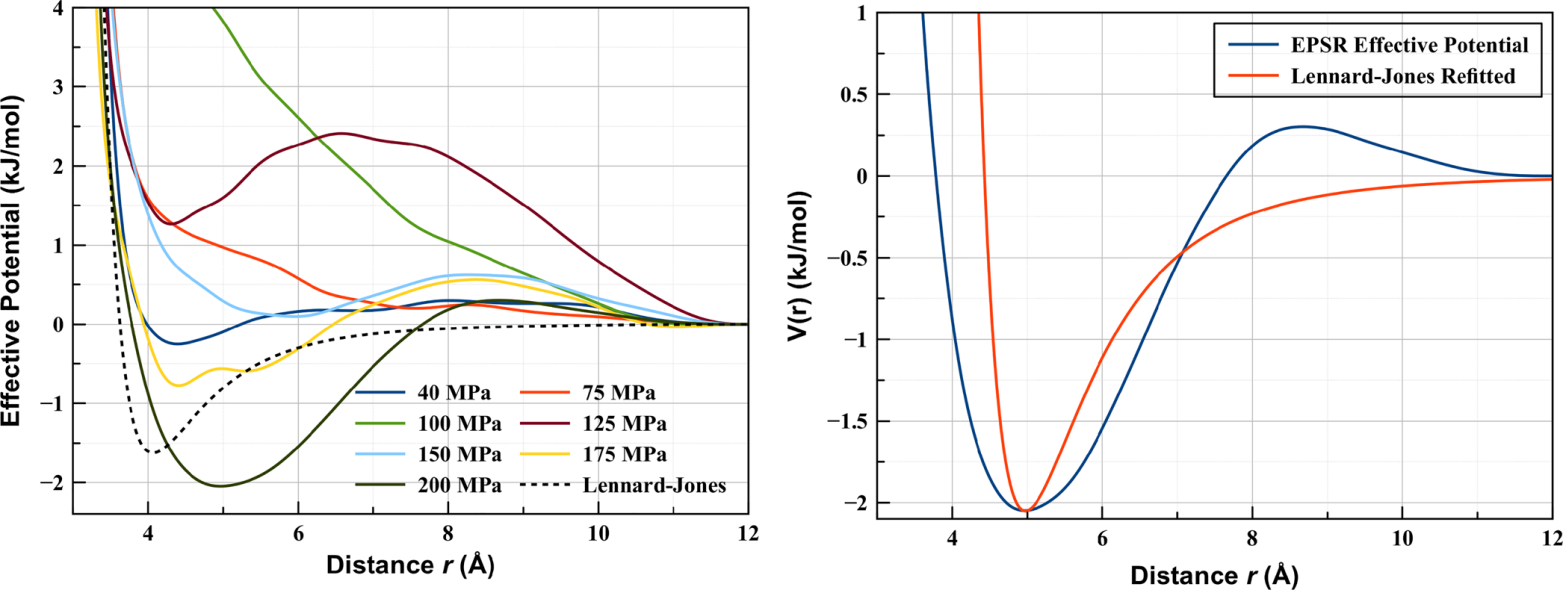
(Left) Effective pairwise interatomic potentials for krypton at all pressures investigated. (Right) Effective potential from EPSR at 200 MPa and refitted Lennard-Jones potential for comparison. The L-J was fitted so that the minimum coincides with the minimum of the effective potential.

Put together, the two situations paint a picture that the Lennard-Jones potential depicts a particle that has both a larger hardcore repulsive volume and is less attractive at higher distances than Kr. This is readily noticeable if we refit the Lennard-Jones potential so that its minimum coincides with that of our EPSR-obtained effective potential (Figure 4, right panel). It is easily visible that there is an 0.65 Å difference in the distance at which the potentials are 0 (the “size of the particle”). This is 3.77 Å for the EPSR potential and 4.42 Å for the refitted Lennard-Jones, resulting in a ∼17% difference in the “size” of a Kr atom if one insists on having a 6–12 analytical form.

Perhaps more important than the quantitative differences noted above, is a fundamental, qualitative one. For all pressures considered in the present study (Figure 4), the effective potential appears to have an almost linear increase with atomic separation *r* beyond the equilibrium separation. The implication is that the attractive forces vary smoothly and slower with increasing distance than Lennard-Jones potentials would indicate.

In the present study we have performed a neutron scattering experiment to determine the *S*(*q*) for krypton at a range of pressures. The amount of structure in the liquid increases with pressure. Up to five oscillations in the *S*(*q*) are detected.

The associated *g*(*r*) is determined in two ways: Fourier transform of *S*(*q*) and EPSR reverse Monte Carlo to find a potential which fits the experimental *S*(*q*). The EPSR method gives an excellent fit to the large-scale structure of the *g*(*r*), while eliminating the high frequency noise which is, presumably, an artifact of the finite range of the experimental *S*(*q*).

The EPSR and experimental (direct Fourier transform) *g*(*r*)’s are in excellent agreement - however the EPSR is unable to reproduce all the oscillations in *S*(*q*): these manifest as rapid oscillations in *g*(*r*), which are clearly unphysical. Ignoring those, it is curious that the fit, which is done in *q*-space, appears more similar to the data in real space (compare Supporting Information Figures 1–4 with Supporting Information Figures 5–8). We conclude that it is impossible for a smoothly varying potential to produce large, high frequency oscillations in *S*(*q*): these are artifacts of the process of Fourier transformation of the data. This situation reinforces the need for EPSR data refinements to be augmented by other theoretical or experimental techniques in order for an accurate and complete picture of a physical system of interest to be obtained. Even for a very simple model system such as krypton, simple insights such as the requirement of a smooth potential strongly impacts how one should interpret *S*(*q*) and by implication the system’s structure, properties, and behavior.

Krypton, with its van der Waals bonding, is regarded as the classical example of the applicability of the Lennard-Jones potential. Its elastic moduli increase proportional to the pressure.^20^ The canonical marker of a system described by pairwise force is the Cauchy pressure^21^ (*C*_12_ – *C*_44_ – 2*P*): in krypton this only diverges from zero above 20 GPa.^20,22^

The excellent fit of the EPSR method to the *g*(*r*) data suggests that a pairwise model for interatomic forces is sufficient to explain the data.

Nevertheless, simulation with a Lennard-Jones potential produces highly overstructured liquids, and the experimental data can only be reproduced by a potential with a softer core than implied by *r*^–12^ and a shallower minimum. These issues apply not just to the standard LJ parametrization by Rutkai et al.^9^ fitted to experimental measurements of vapor pressure, saturated liquid density, and the critical temperature and density but also to a rescaled LJ with the energy minimum optimized to the current data. We conclude that the bonding in supercritical Kr is not well described by any potential in the Lennard-Jones form.

A simulation of krypton using density functional theory shows similar *g*(*r*) and shows that a van der Waals correction also serves to overstructure the liquid (see Supporting Information).

A notable feature of the *g*(*r*) is the well-defined second peak and discernible third peak. These are located at approximately two and three times the radius of the first peak: much further out than would be found in a crystal structure.

This has allowed us to determine that the Frenkel line in Kr crosses the room temperature isotherm at (310 K/110 MPa). This is done using the Pruteanu–Ackland criteria based on the coordination number.^4^ The temperature (310 K ∼ 1.5*T*_*C*_, critical temperature *T*_*C*_ = 209.46 K) and pressure (110 MPa ∼ 20*P*_*C*_, critical pressure *P*_*C*_ = 5.52 MPa)^23^ are in very good agreement with theoretical predictions by Brazhkin et al.^11^ for the location of the Frenkel line.

## Supporting Information Available

Measured *S*(*q*)’s and EPSR fits; raw Fourier transformed *G*(*r*), EPSR fitted, and Lennard-Jones *g*(*r*)’s; all EPSR residuals and *R*-factors; running coordination numbers from EPSR; effect of Lennard-Jones parameters; *S*(*q*) ’s and EPSR fits Constraining the empirical potential amplitude; EPSR pair distribution functions with constrained empirical potential amplitude; effect of empirical potential amplitude on EPSR Fit; comparison with Teitsma data on low pressure Kr; and density functional theory calculations (PDF)
