# Supplementary material for: Krypton and the Fundamental Flaw of the Lennard-Jones Potential
Source: J Phys Chem Lett. 2022 Aug 29;13(35):8284–9. doi: 10.1021/acs.jpclett.2c02004 (PMC9465677; doi:10.1021/acs.jpclett.2c02004)
Supplement: Supplementary file 1 — jz2c02004_si_001.pdf [file jz2c02004_si_001.pdf]

Krypton and the Fundamental Flaw of the Lennard-Jones Potential

SUPPORTING INFORMATION

Ciprian G. Pruteanu,[*,†] John S. Loveday,[†] Graeme J. Ackland,[*,†] and John E. Proctor[*,‡]

†*SUPA, School of Physics and Astronomy and Centre for Science at Extreme Conditions, The University of Edinburgh, Edinburgh EH9 3FD, United Kingdom*

‡*Materials & Physics Research Group, Newton Building, University of Salford, Manchester M5 4WT, United Kingdom*

E-mail: cip.pruteanu@ed.ac.uk; gjackland@ed.ac.uk; j.e.proctor@salford.ac.uk

Contents

This document contains:

- Measured $S(q)$'s and EPSR Fits
- Raw Fourier transformed $G(r)$, EPSR fitted, and Lennard-Jones $g(r)$'s
- All EPSR Residuals and R-Factors
- Running Coordination Numbers from EPSR
- Effect of Lennard-Jones Parameters
- $S(q)$'s and EPSR Fits Constraining the Empirical Potential Amplitude
- EPSR Pair Distribution Functions with Constrained Empirical Potential Amplitude
- Effect of Empirical Potential Amplitude on EPSR Fit
- Comparison with Teitsma Data on Low Pressure Kr
- Density Functional Theory Calculations

Measured $S(q)$'s and EPSR Fits

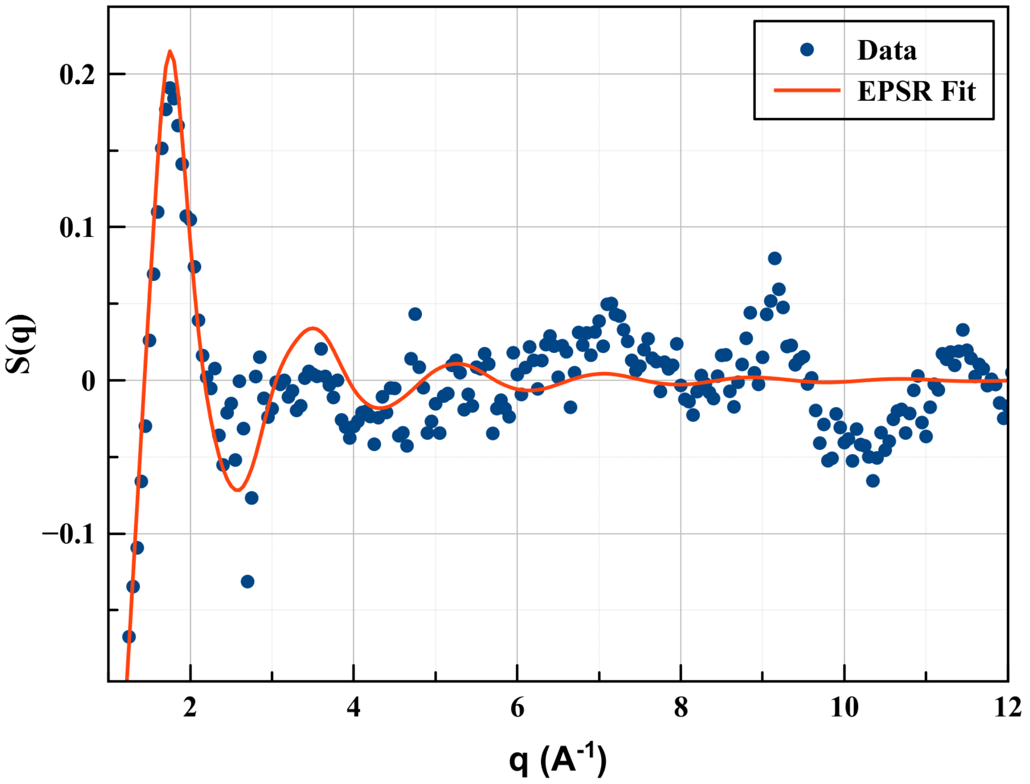

Figure 1: Measured $S(q)$ and EPSR fit for 40 MPa dataset.

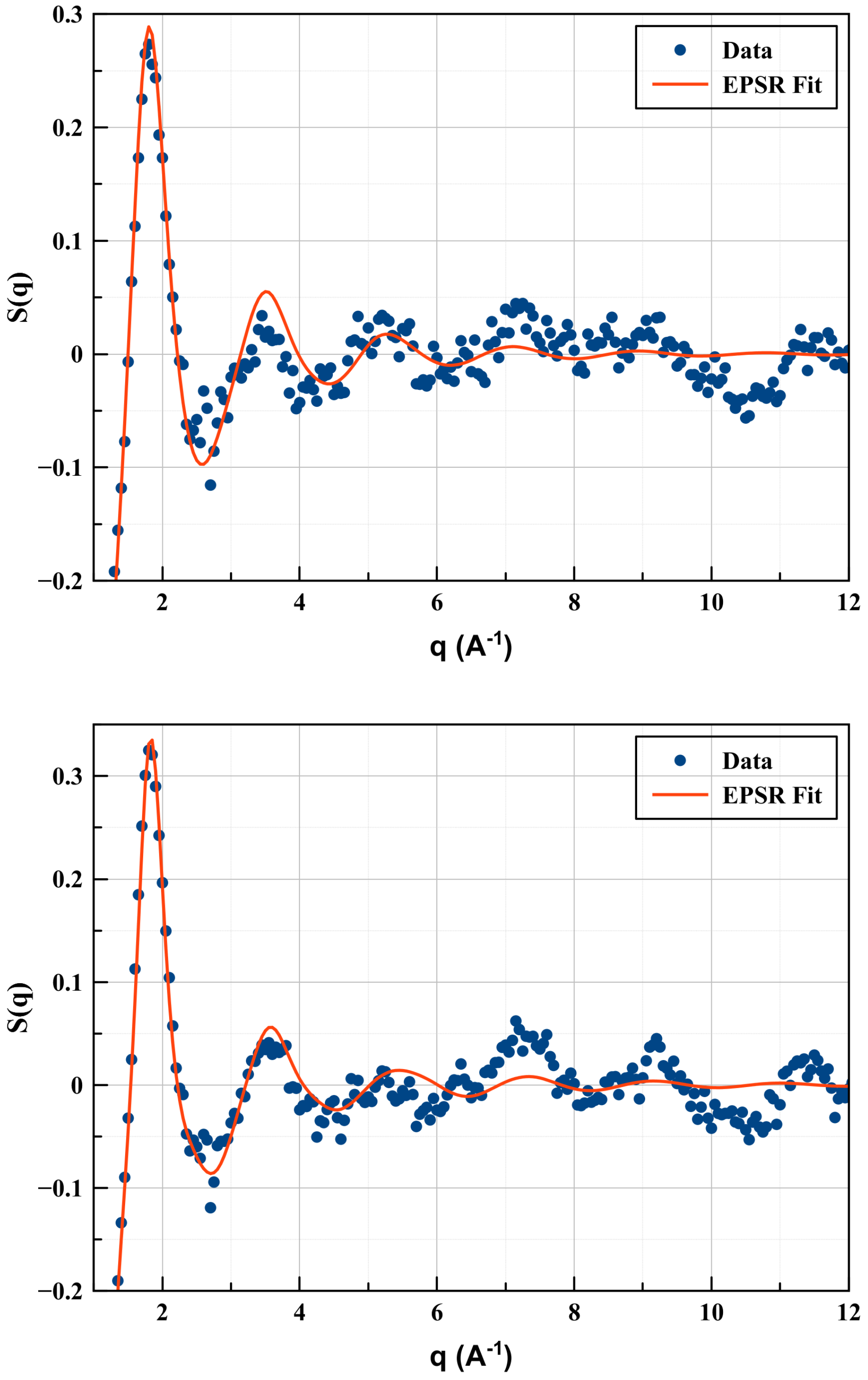

Figure 2: (Top) Measured $S(q)$ and EPSR fit for 75 MPa. (Bottom) Measured $S(q)$ and EPSR fit for 100 MPa.

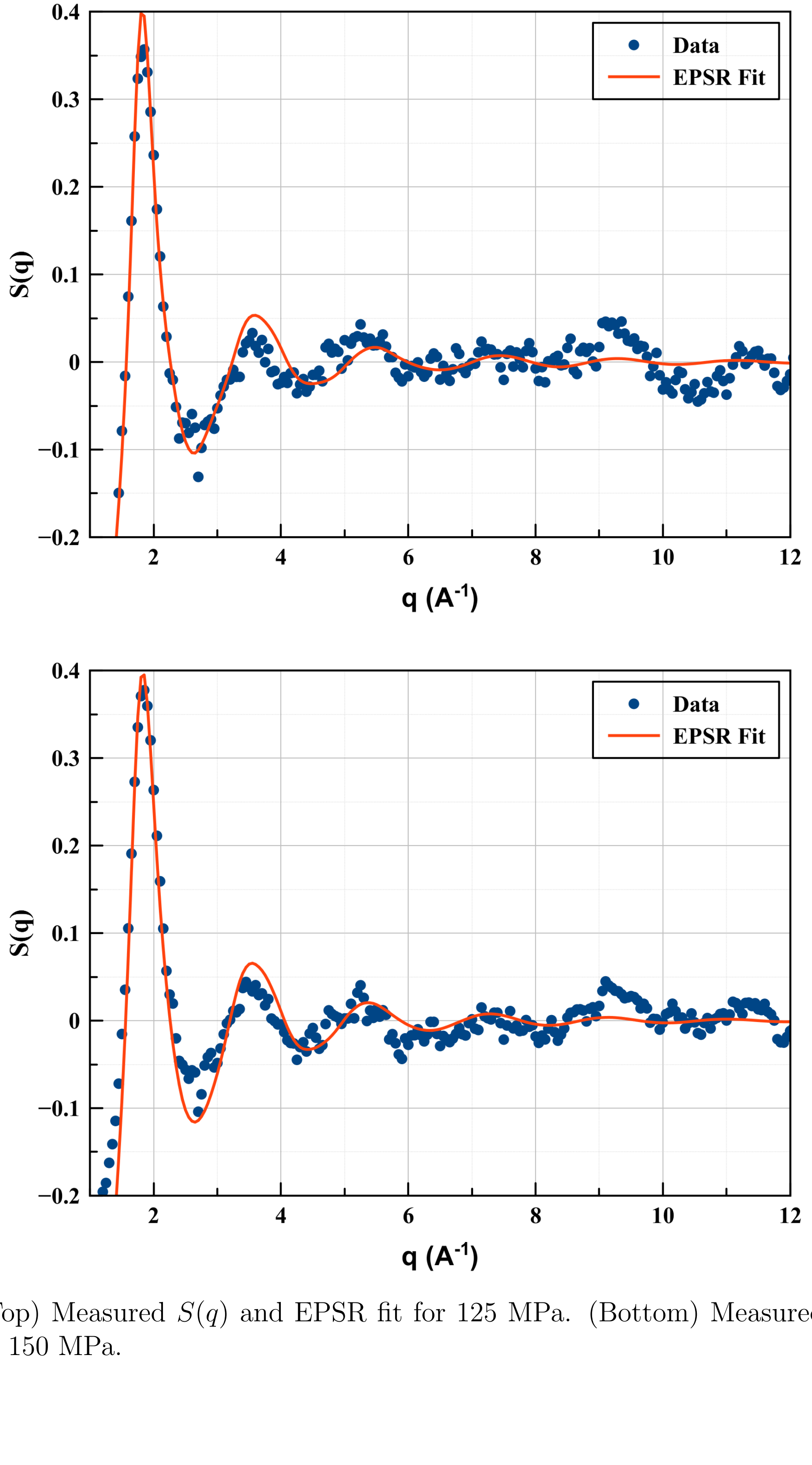

Figure 3: (Top) Measured $S(q)$ and EPSR fit for 125 MPa. (Bottom) Measured $S(q)$ and EPSR fit for 150 MPa.

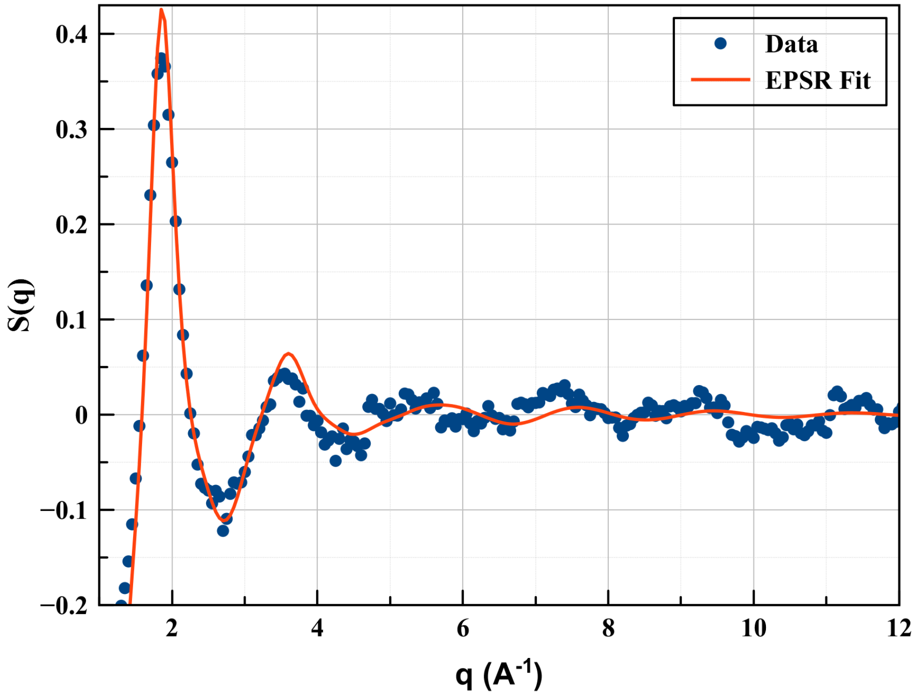

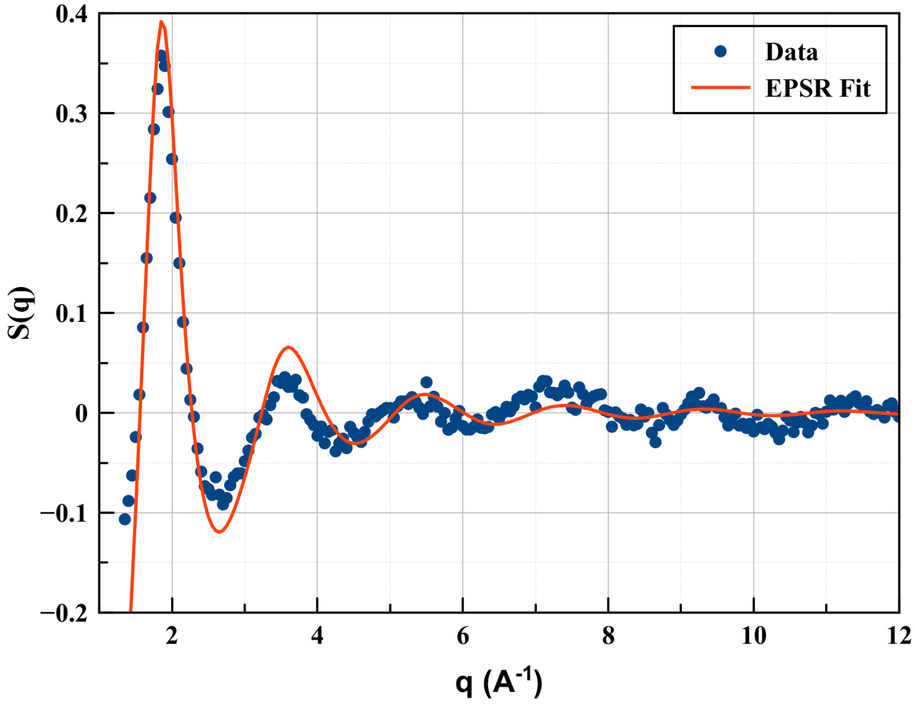

Figure 4: (Top) Measured $S(q)$ and EPSR fit for 175 MPa. (Bottom) Measured $S(q)$ and EPSR fit for 200 MPa.

Raw Fourier Transformed $G(r)$, EPSR Fitted $g(r)$'s and Monte Carlo Lennard-Jones $g(r)$'s

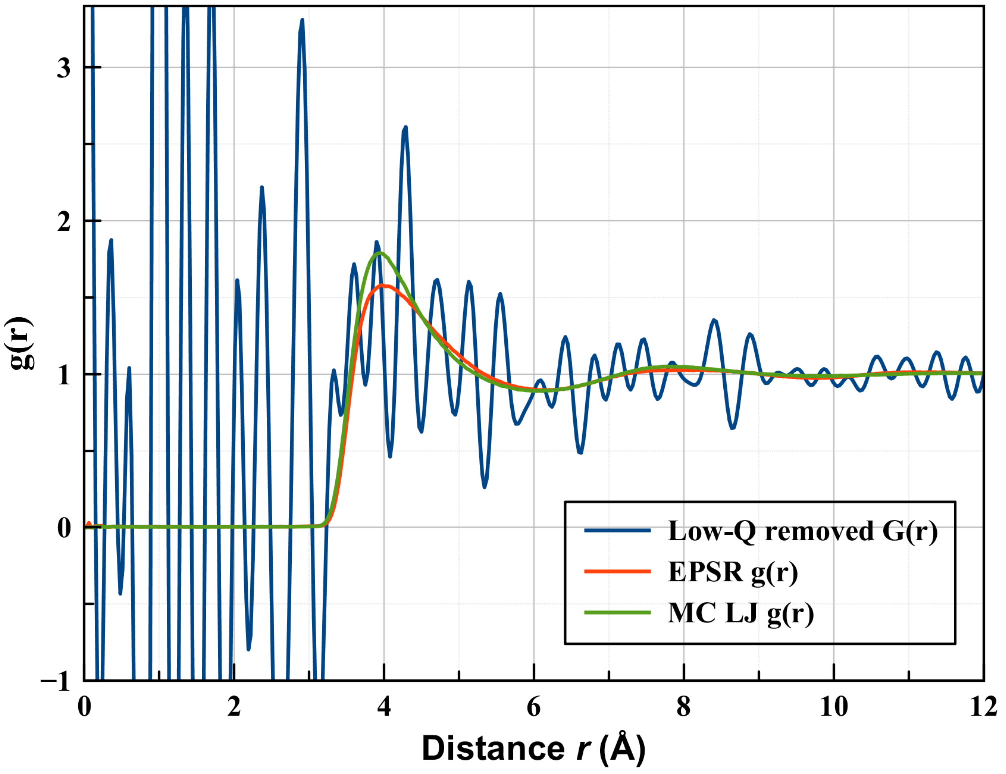

Figure 5: Raw Fourier transformed $G(r)$, Monte Carlo Lennard-Jones $g(r)$ and EPSR fit $g(r)$ for 40 MPa dataset.

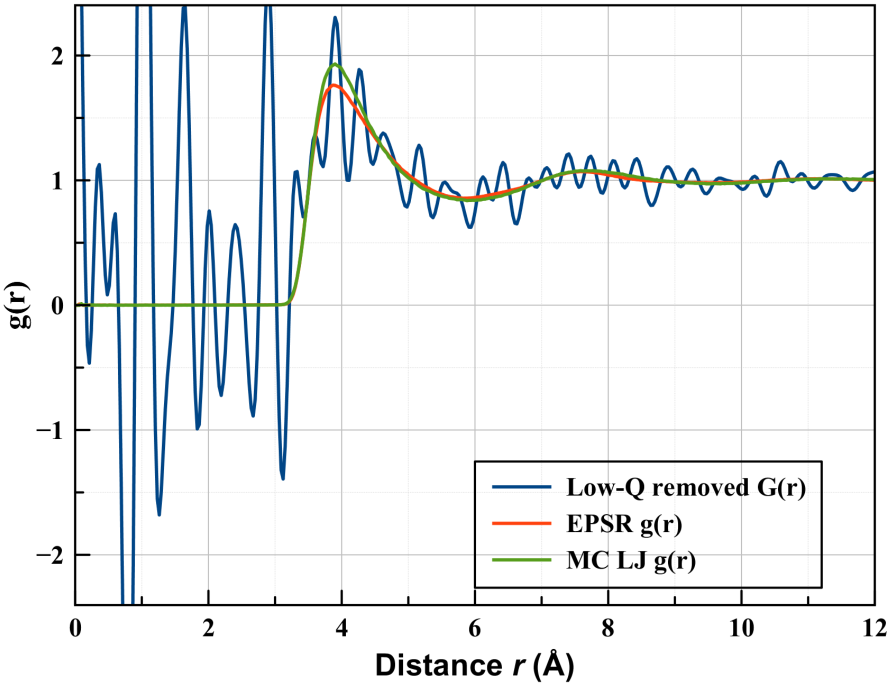

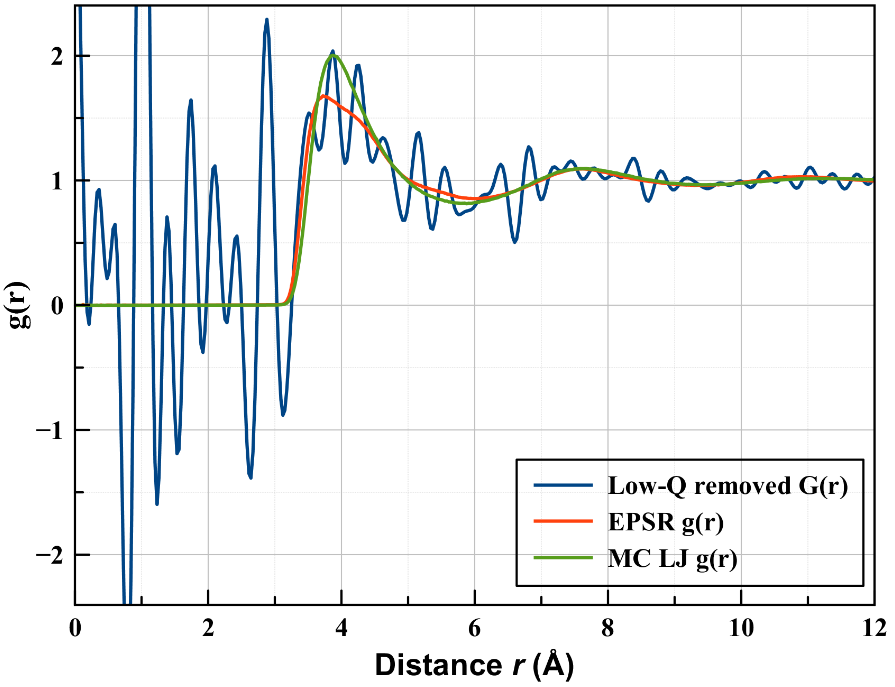

Figure 6: (Top) Raw Fourier transformed $G(r)$, Monte Carlo Lennard-Jones $g(r)$ and EPSR fit $g(r)$ for 75 MPa. (Bottom) Raw Fourier transformed $G(r)$, Monte Carlo Lennard-Jones $g(r)$ and EPSR fit $g(r)$ for 100 MPa.

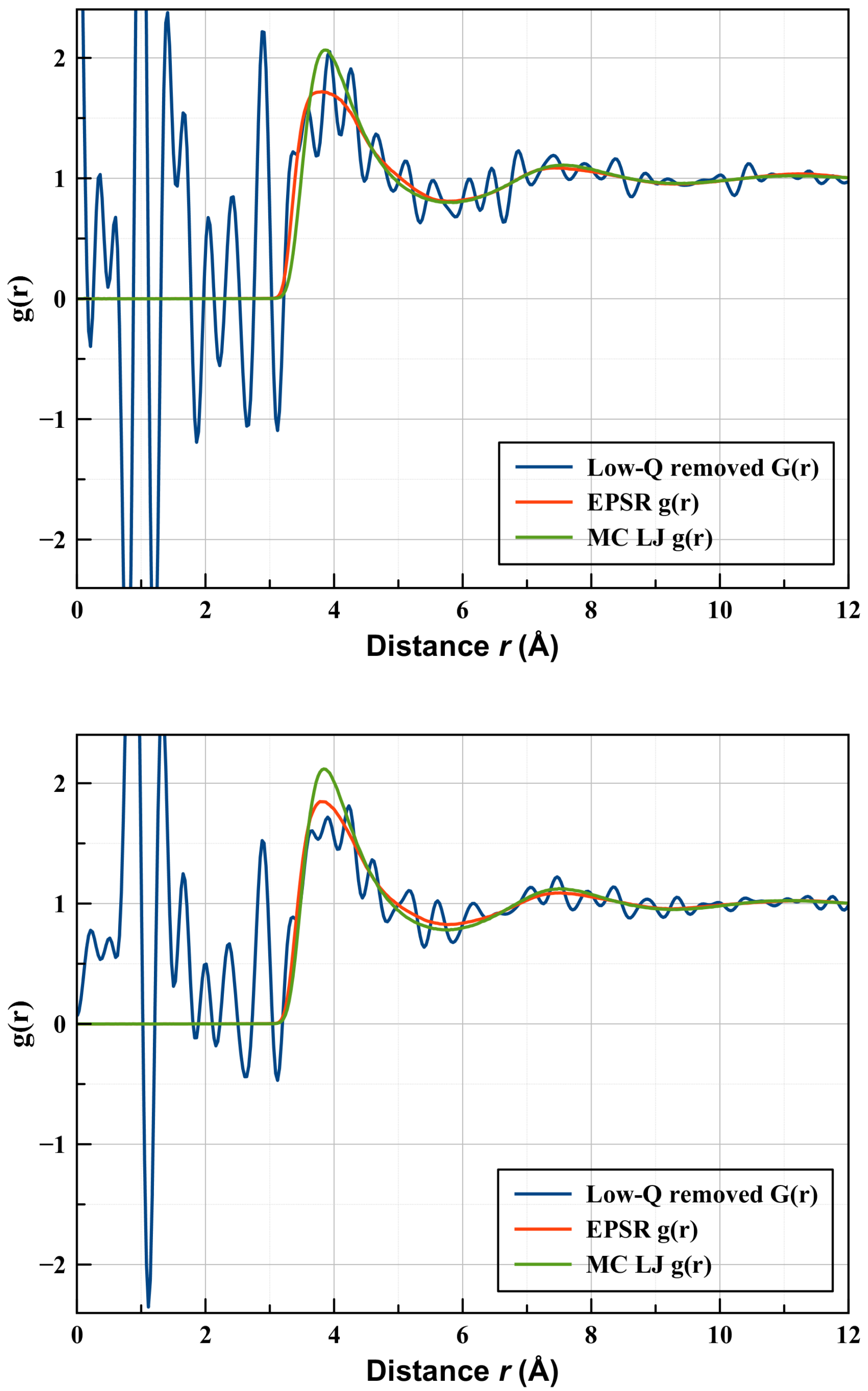

Figure 7: (Top) Raw Fourier transformed $G(r)$, Monte Carlo Lennard-Jones $g(r)$ and EPSR fit $g(r)$ for 125 MPa. (Bottom) Raw Fourier transformed $G(r)$, Monte Carlo Lennard-Jones $g(r)$ and EPSR fit $g(r)$ for 150 MPa.

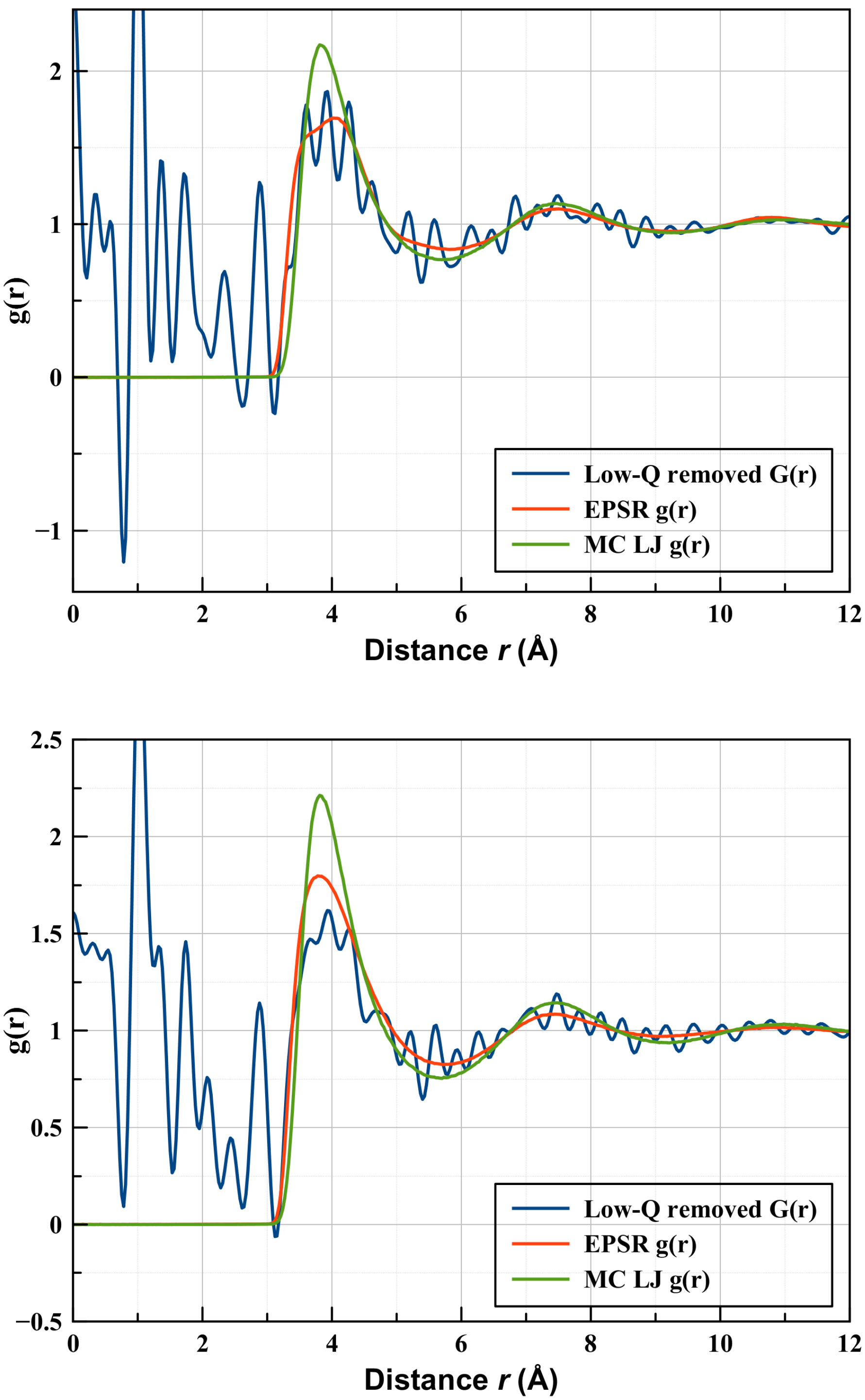

Figure 8: (Top) Raw Fourier transformed $G(r)$, Monte Carlo Lennard-Jones $g(r)$ and EPSR fit $g(r)$ for 175 MPa. (Bottom) Raw Fourier transformed $G(r)$, Monte Carlo Lennard-Jones $g(r)$ and EPSR fit $g(r)$ for 200 MPa.

All EPSR Residuals and R-Factors

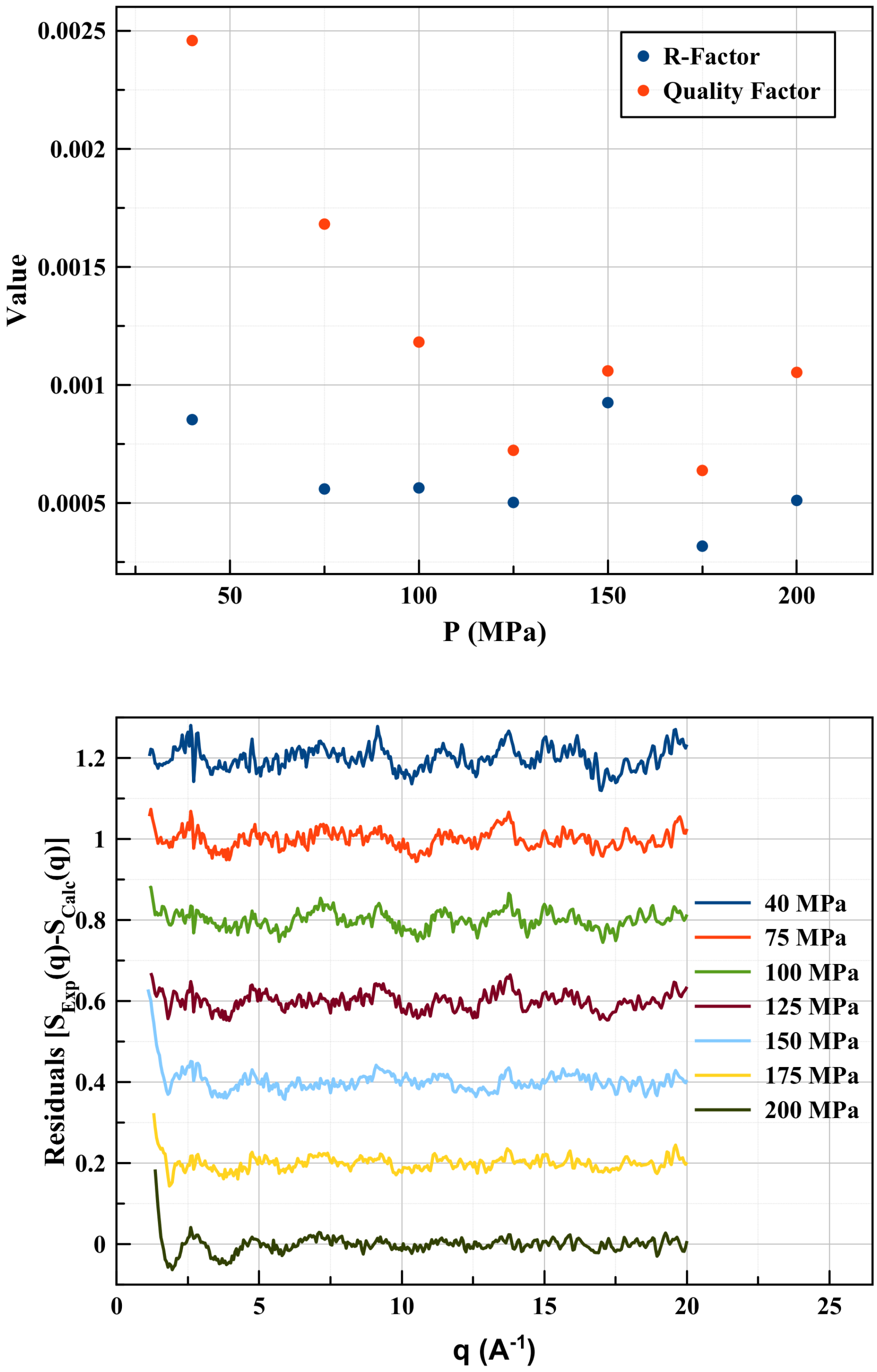

Figure 9: (Top) All EPSR R-factors and Quality Factors for the datasets in the present study. (Bottom) Residuals for all the datasets fitted in the present study.

Running Coordination Numbers from EPSR

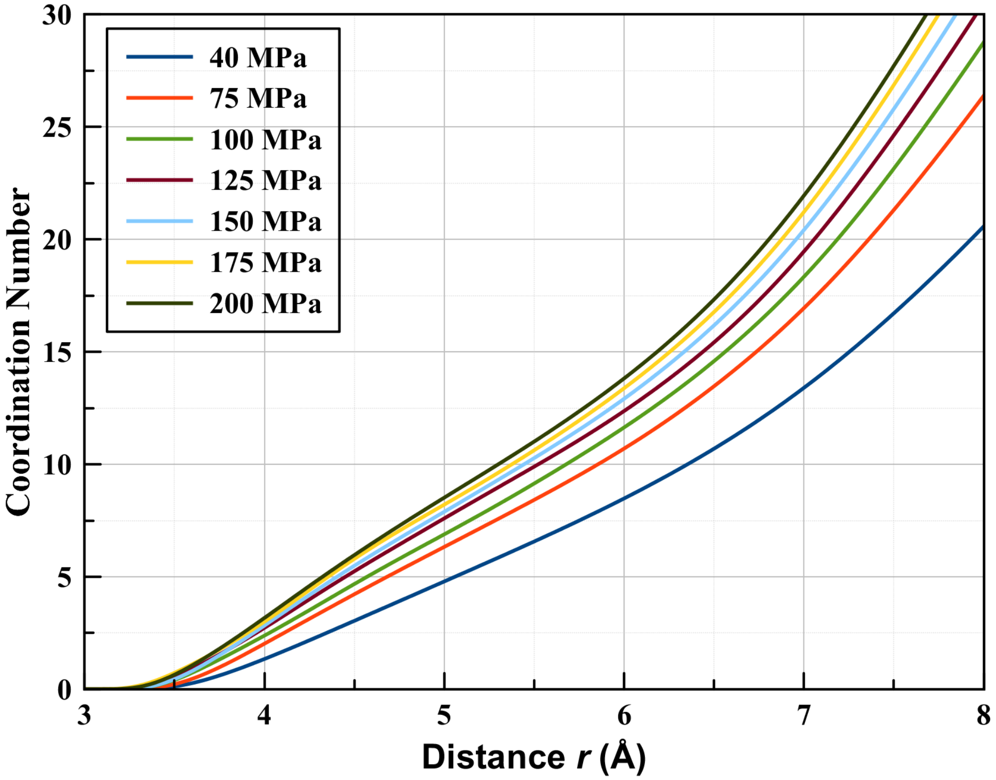

Figure 10: All EPSR calculated running coordination numbers for the datasets presented in the current study.

Effect of Lennard-Jones Parameters

Monte Carlo fits were performed to the data with 2 different parametrizations of the Lennard-Jones potential. One using the values of Rutkai et al. (ϵ=1.69 kJ/mol ,σ=3.62 Å) and one using ϵ=1.39 kJ/mol and σ=3.6 Å.

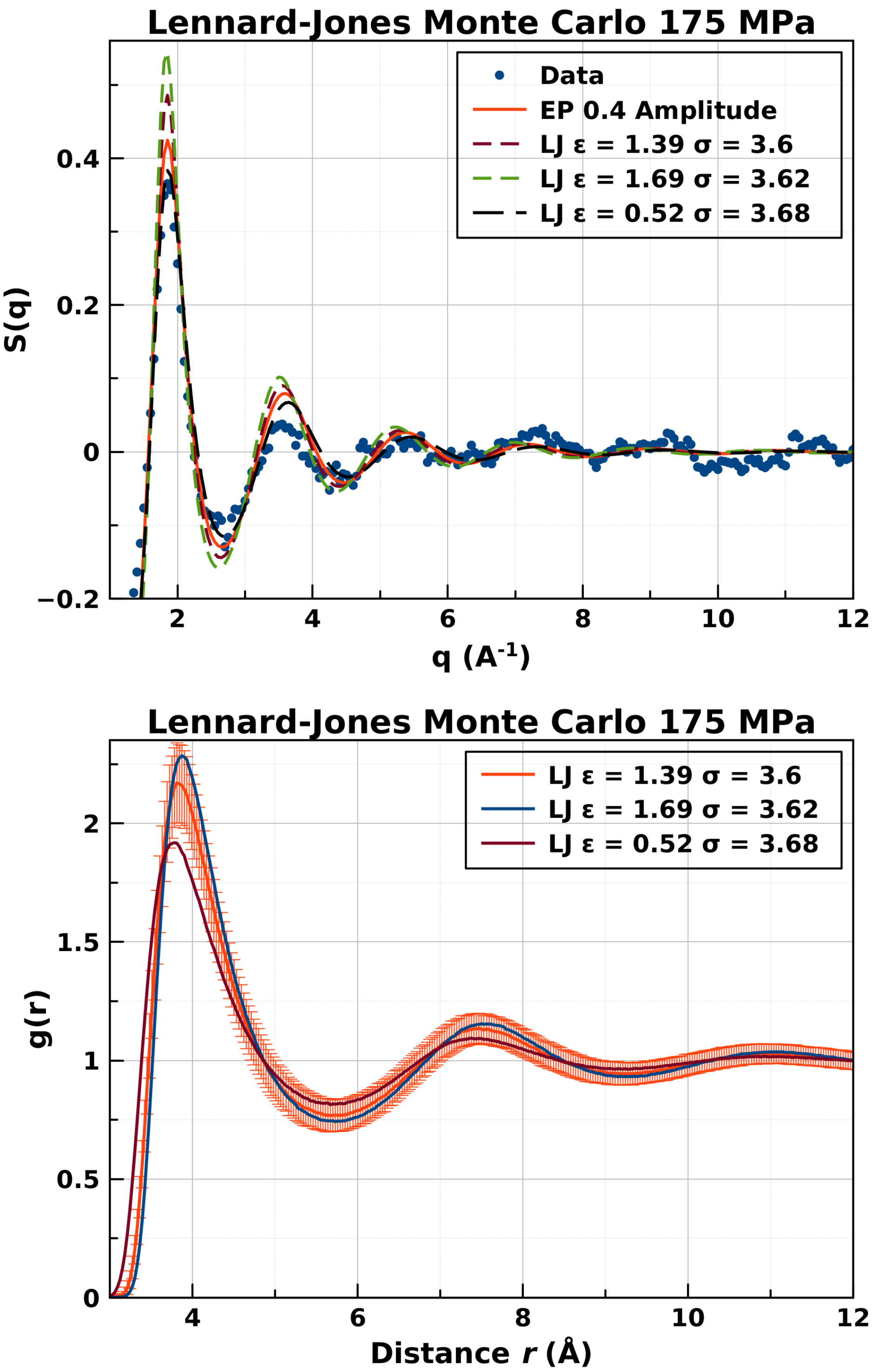

Figure 11: Data at 175 MPa and Monte Carlo fits of 2 different Lennard-Jones sets of parameters. (Top) $S(q)$'s and fits (Bottom) Extracted pair distribution functions.

S(q)'s and EPSR Fits Constraining the Empirical Potential Amplitude

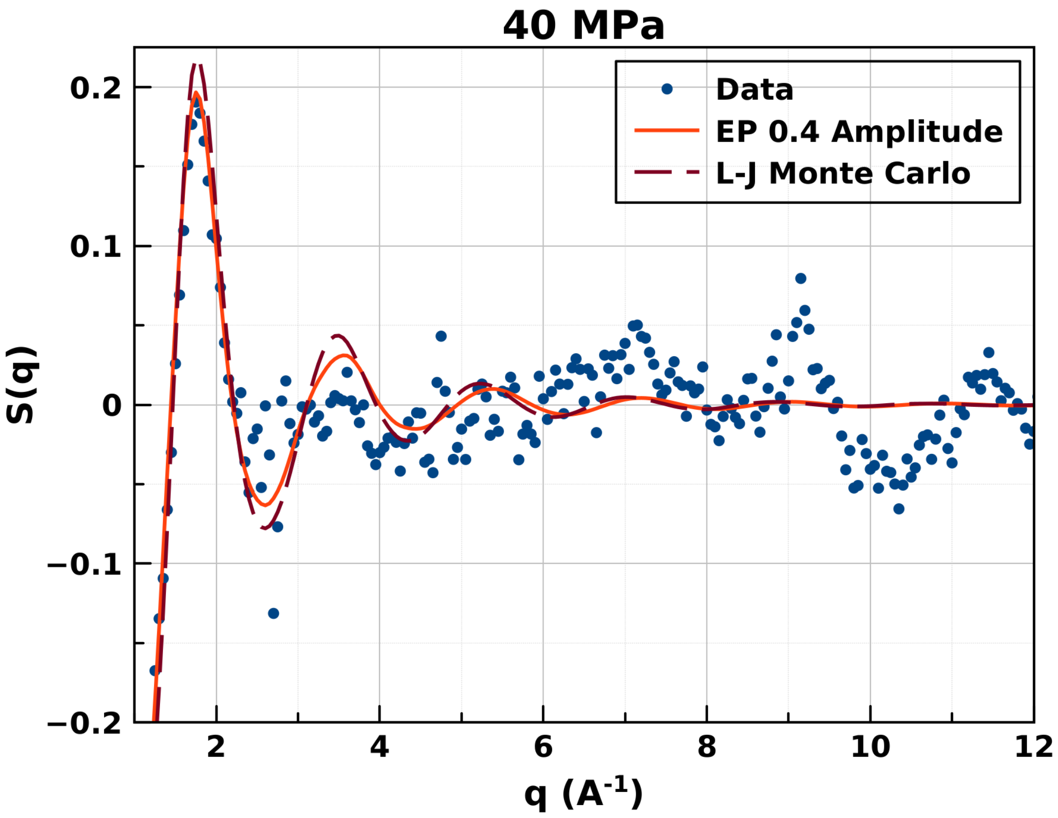

Figure 12: Measured $S(q)$, EPSR fit with 0.4 Empirical Potential (EP) Amplitude and pure Lennard-Jones Monte Carlo for the same density box.

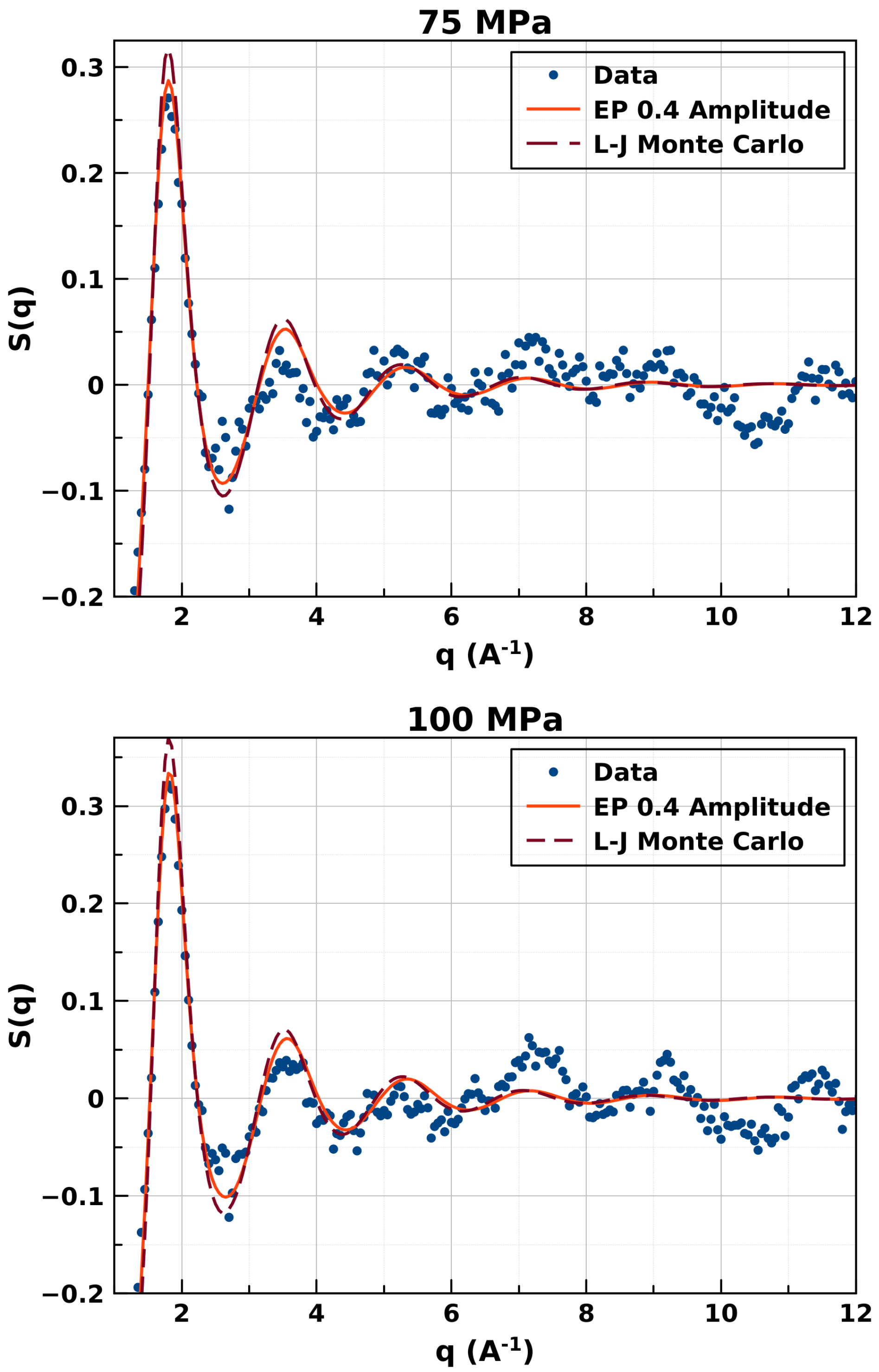

Figure 13: Measured $S(q)$, EPSR fit with 0.4 Empirical Potential (EP) Amplitude and pure Lennard-Jones Monte Carlo for the same density box.

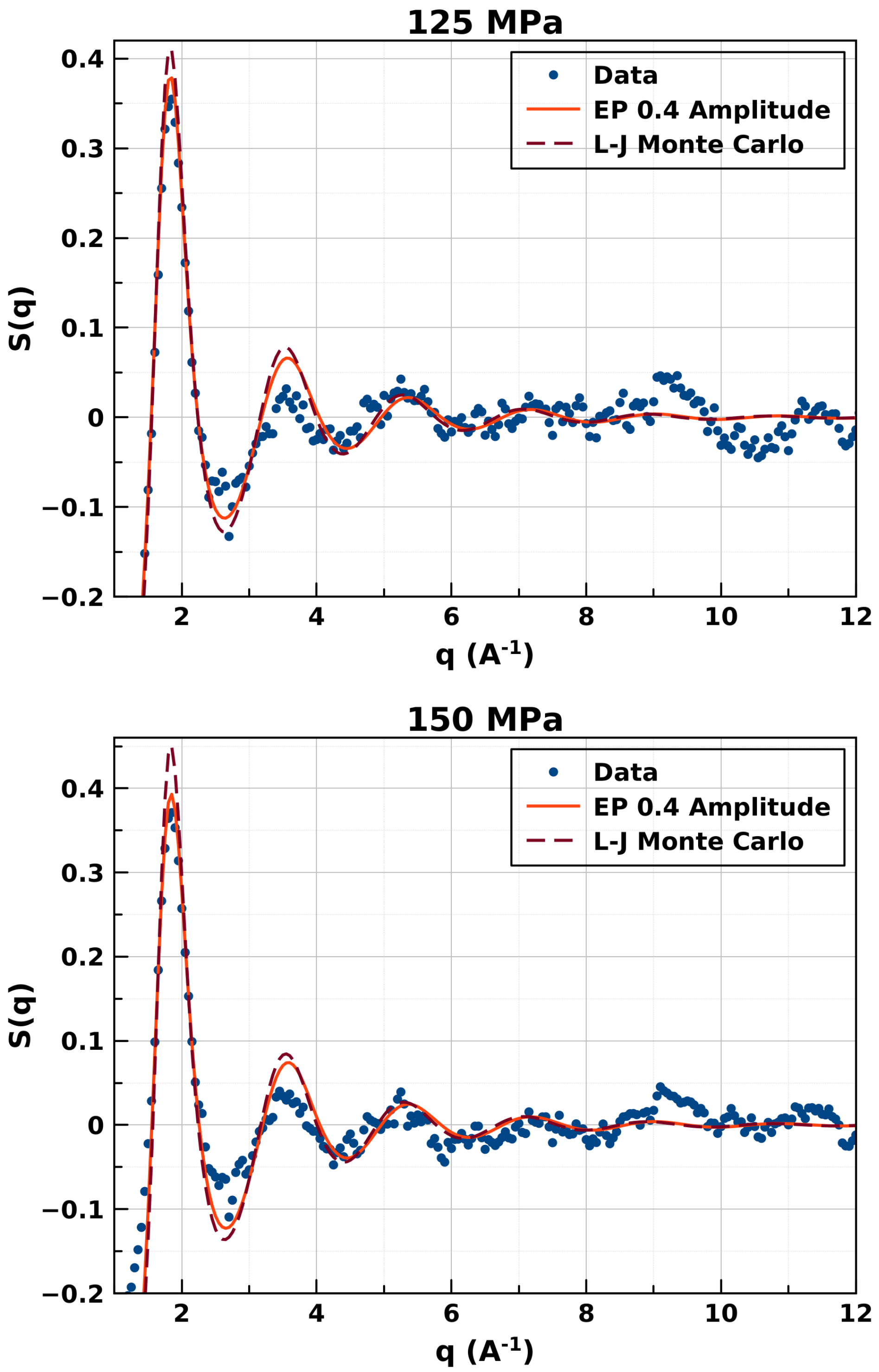

Figure 14: Measured $S(q)$, EPSR fit with 0.4 Empirical Potential (EP) Amplitude and pure Lennard-Jones Monte Carlo for the same density box.

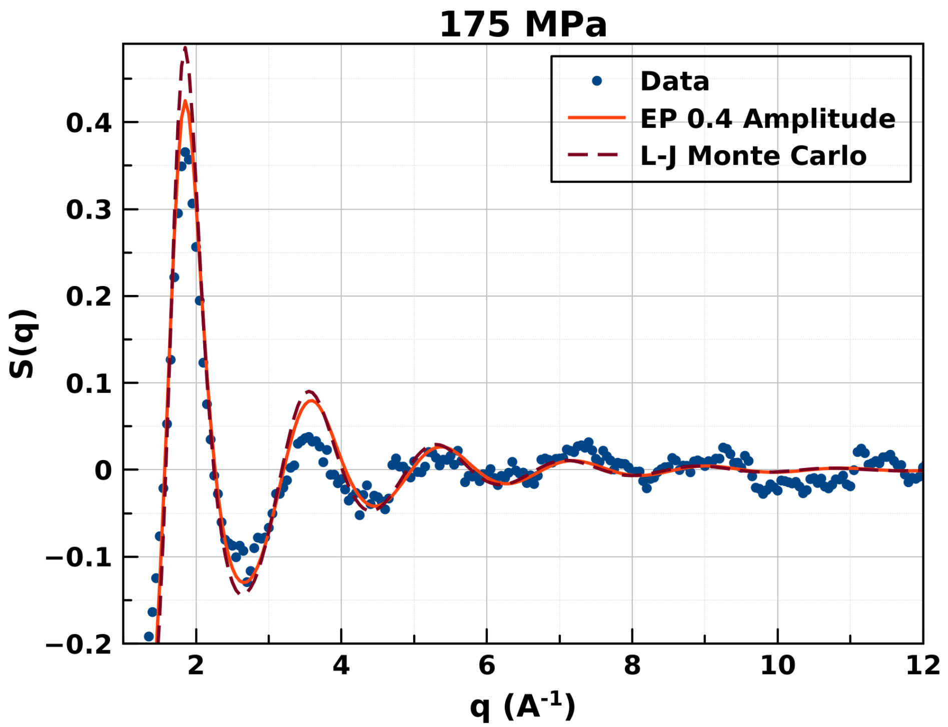

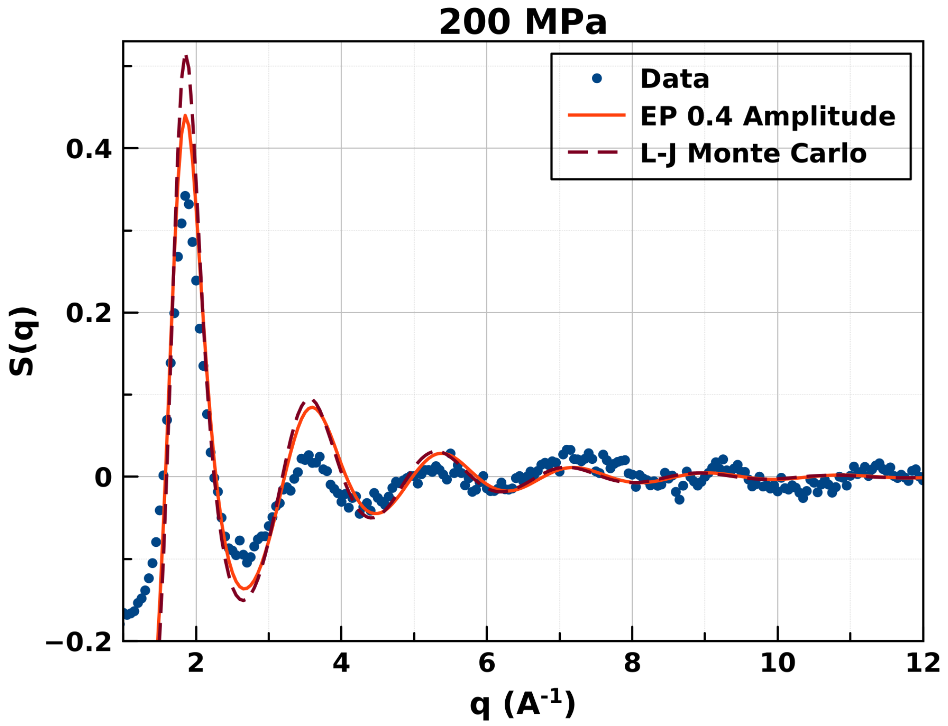

Figure 15: Measured $S(q)$, EPSR fit with 0.4 Empirical Potential (EP) Amplitude and pure Lennard-Jones Monte Carlo for the same density box.

EPSR Pair Distribution Functions with Constrained Empirical Potential Amplitude

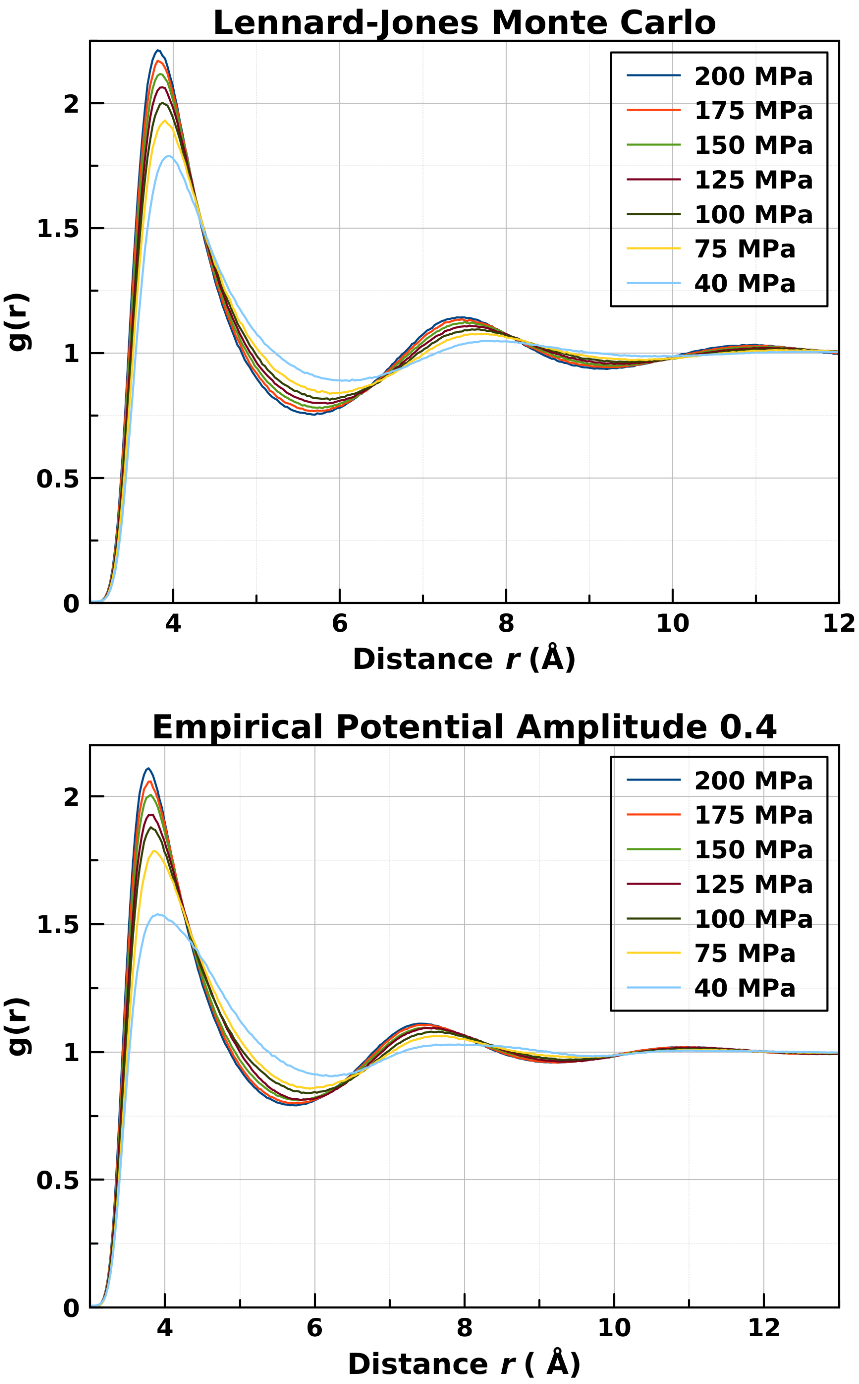

Figure 16: All $g(r)$'s from pure Lennard-Jones Monte Carlo simulations (top) and EPSR fit with 0.4 Empirical Potential Amplitude (bottom).

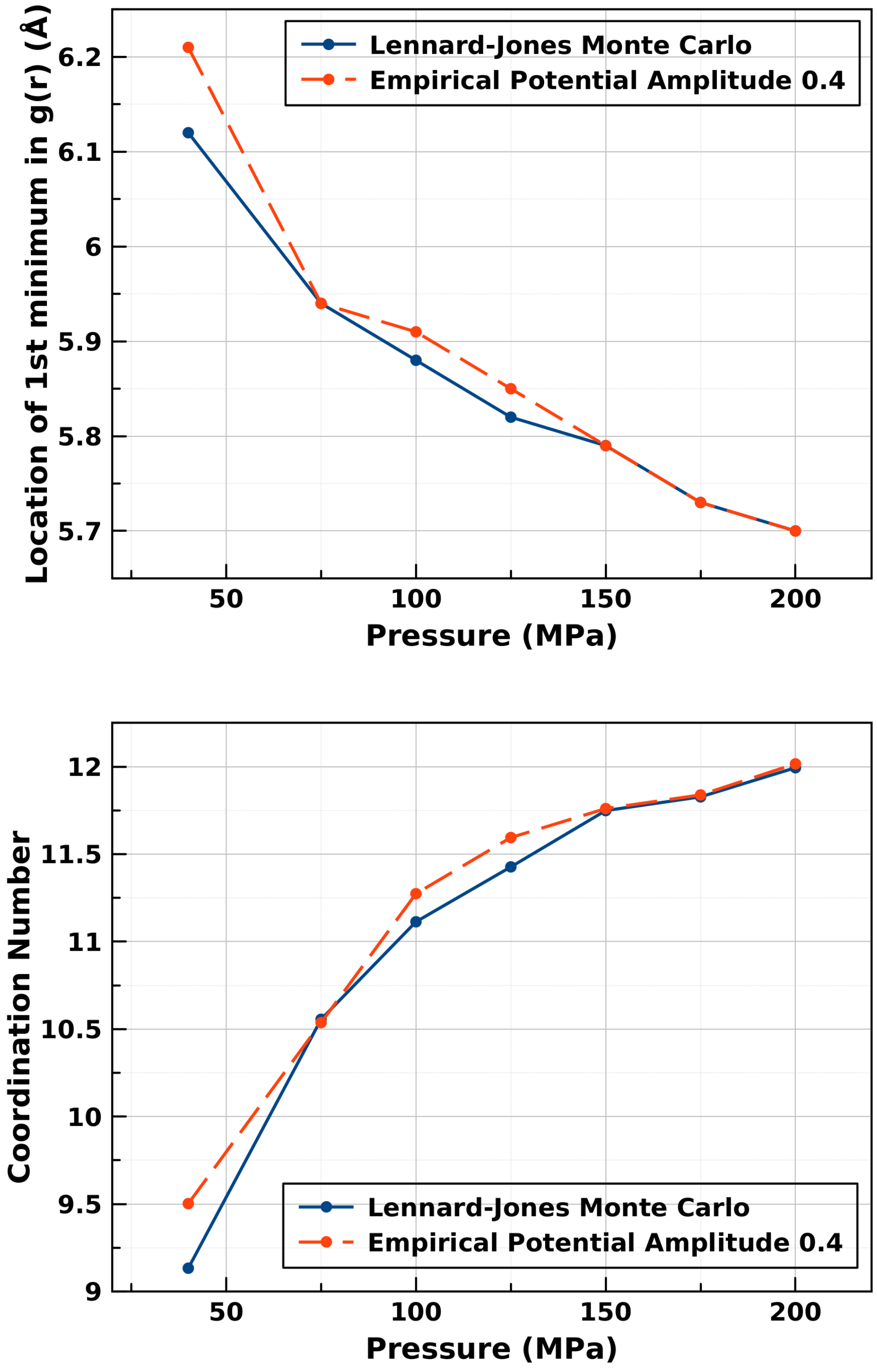

Figure 17: Size of the first coordination shell (top) and comparison between coordination numbers from Lennard-Jones Monte Carlo simulations and EPSR fits with Empirical Potential Amplitude of 0.4 (bottom).

Effect of Empirical Potential Amplitude on EPSR Fit

We have re-fitted the 175 MPa datasets using various fixed amplitudes for the empirical potential and evaluated their impact on the obtained $g(r)$'s and coordination numbers. All results are presented below.

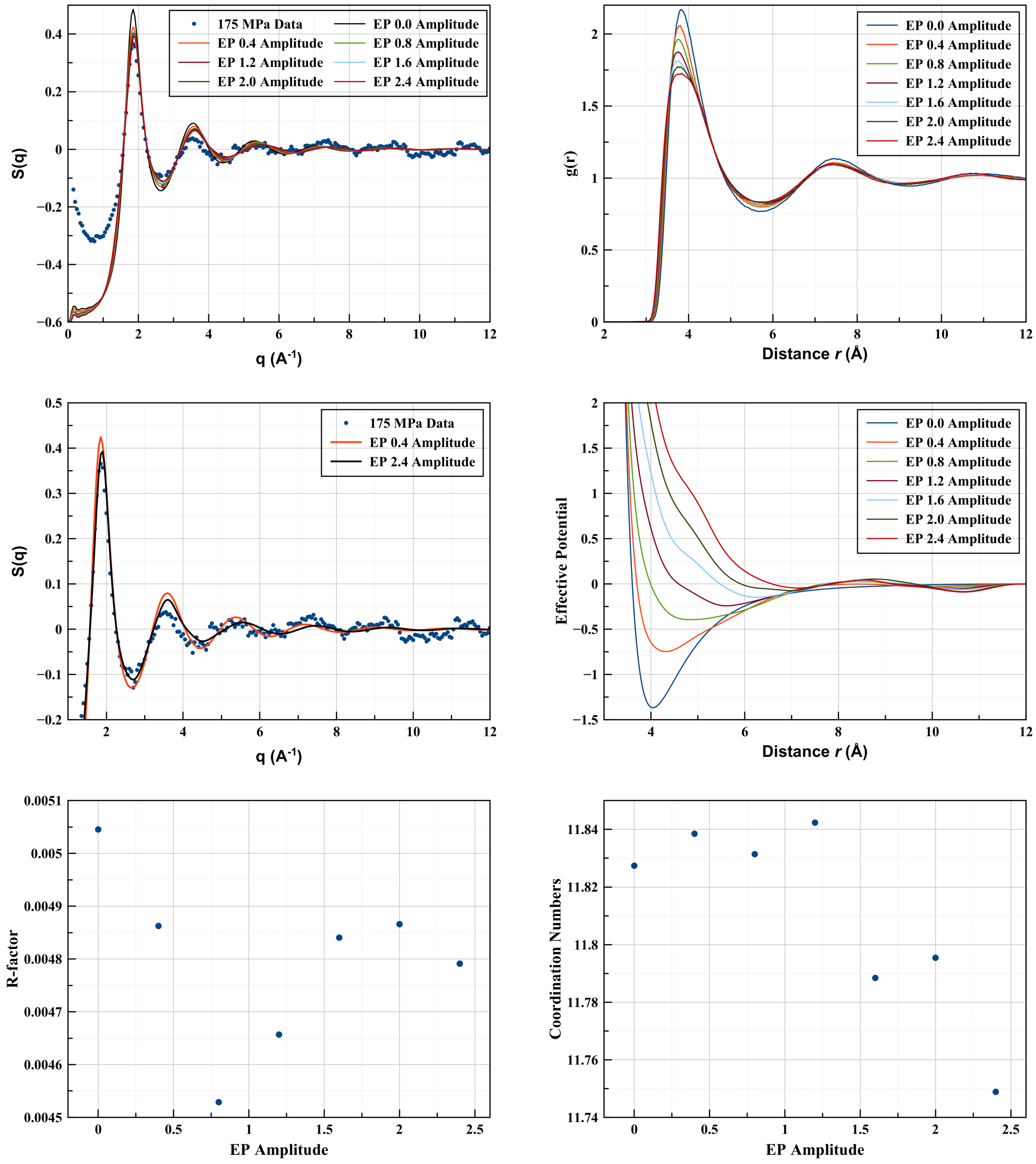

Figure 18: Results of re-fitting the 175 MPa dataset using different values for the empirical potential amplitude. Both R-factor and coordination number are insensitive to the potential amplitude. In the case of R, this is simply because R is dominated by the unphysical low-Q data. The stable coordination number shows that the atoms are in a close-packed liquid, and the dominant term in the free energy comes from PV rather than the potential.

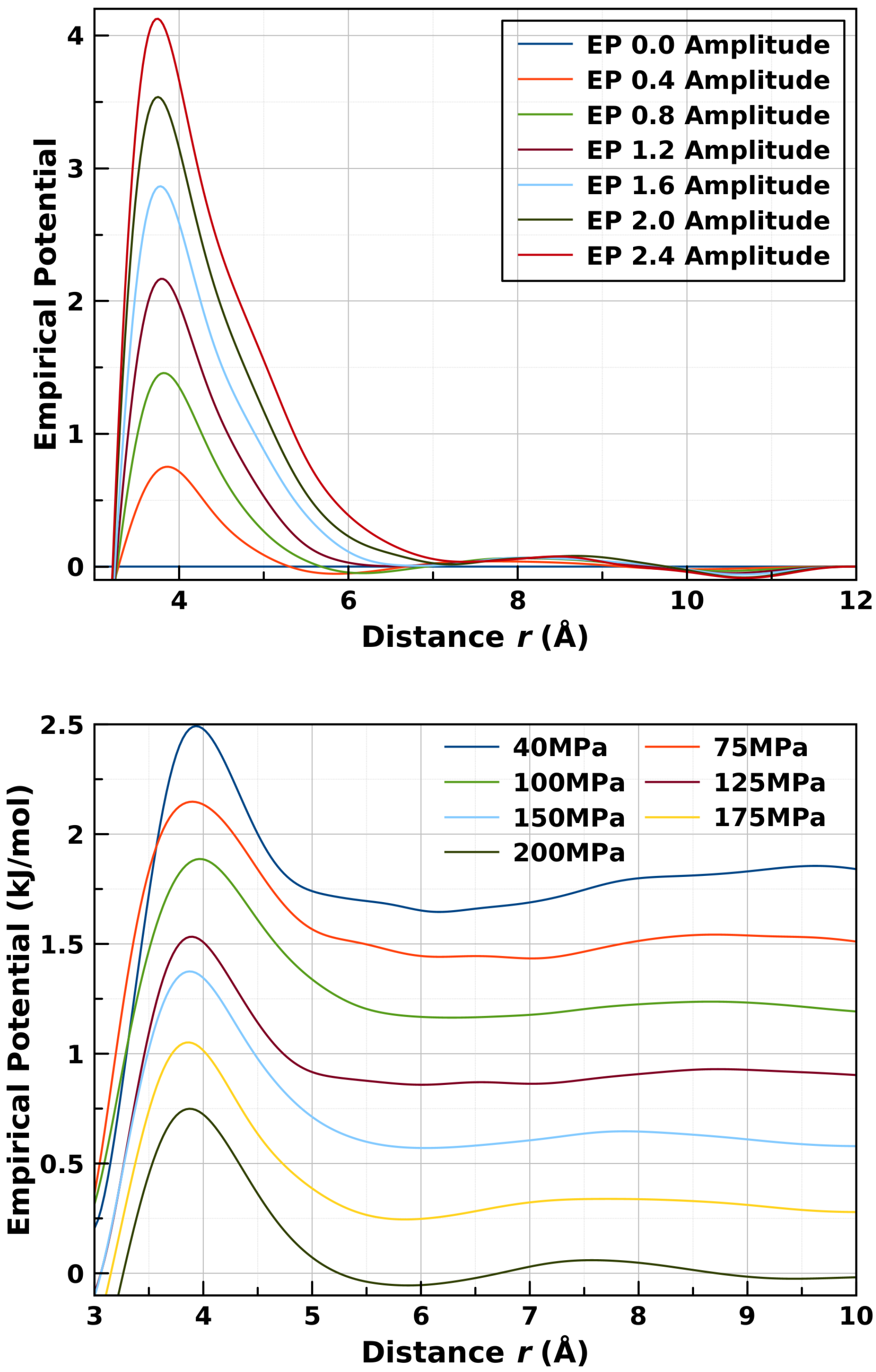

Figure 19: (Top) Variation of the empirical potential with set amplitude for 175 MPa (Bottom) Empirical Potentials with set amplitude of 0.4 for all pressures considered in the present study.

Comparison with Teitsma Data on Low Pressure Kr

Teitsma's data[1] for low pressure Kr was also run through EPSR similarly to the datasets collected for this work. Due to the poor data quality the fits are significantly worse than for our current data, a clear lack of background correction and normalization being easily visible.

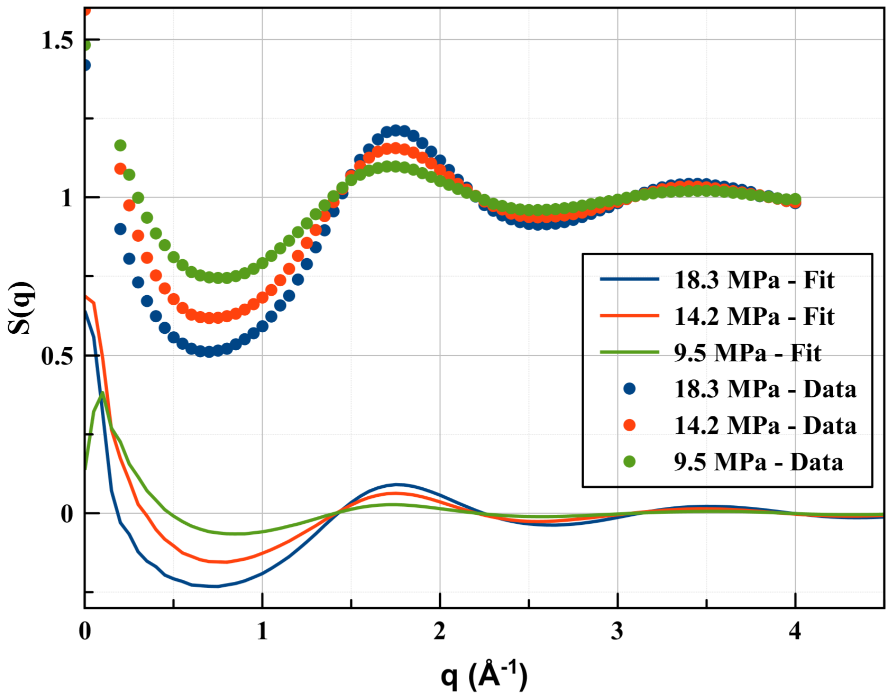

Figure 20: $S(q)$'s as reported by Teitsma for Kr at 3 different low densities/pressure. The lack of appropriate background correction for the data sets is readily visible.

Below, the extracted pair distribution functions and coordination numbers are shown. It is readily noticeable the coordination numbers are in good agreement with the current work and resulting location of the Frenkel line.

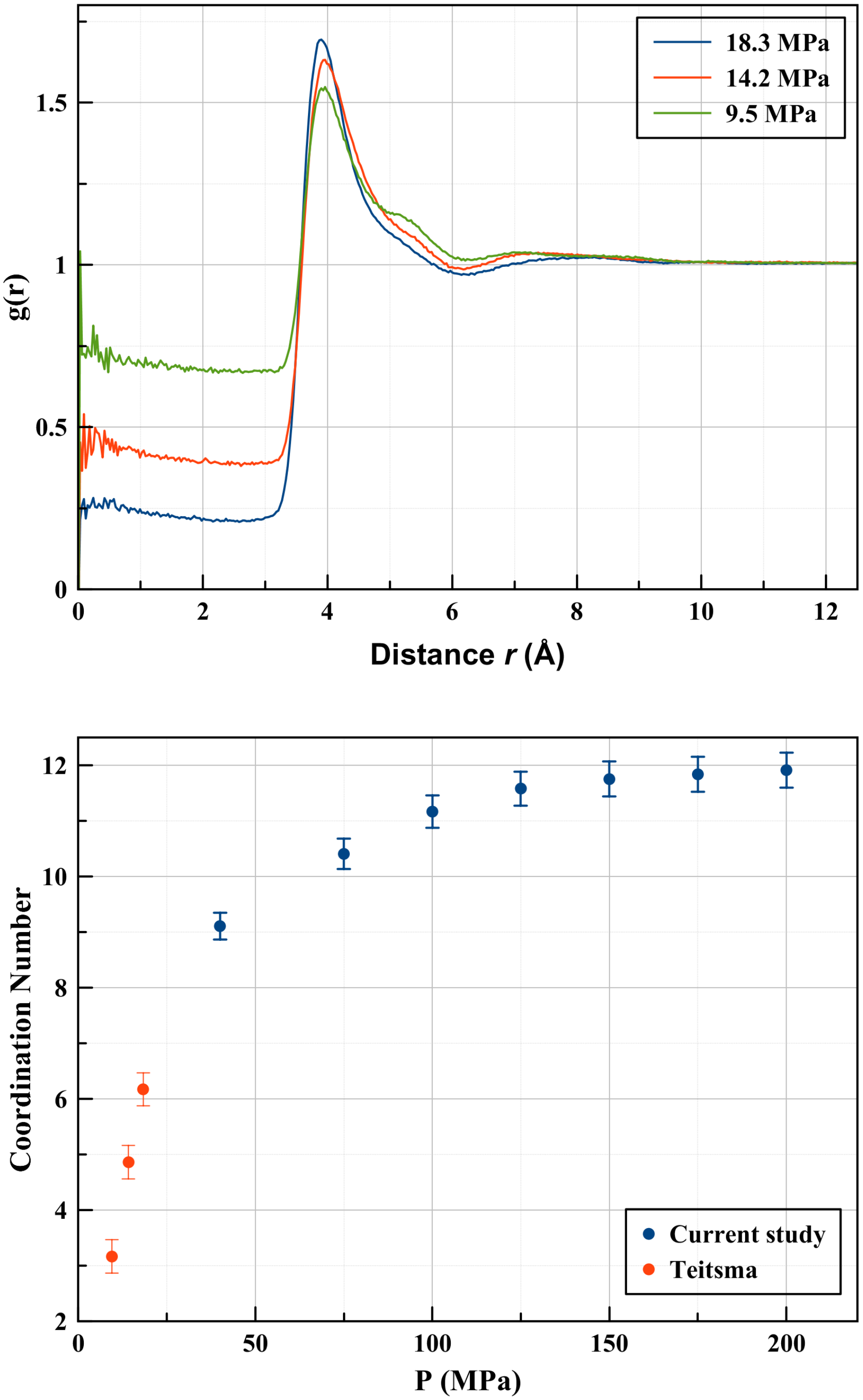

Figure 21: Pair Distribution Functions extracted from EPSR from Teistma's data (top) and extended coordination number vs. pressure curve using Teitsma's and current data (bottom).

Density Functional Theory Calculations

We carried out a series of density functional theory calculations on fluid Krypton using the CASTEP code (version 19.11, standard settings). We used both PBE and PBE plus Tkatchenko-Scheffler (TS) dispersion correction scheme. Ultrasoft pseudopotentials with a 190.5eV plane-wave cut-off for the basis set were employed. The bands are flat and the system is not periodic, so gamma-point sampling was employed. MD was run in the NVT ensemble with a 2fs timestep for up to 10ps with 100 atoms. Five densities were investigated with volume per atom 40, 70, 100, 200, and 400 Å^3. The associated Radial Distribution Functions are shown in Figure 22. The highest density - well above those reached in experiment - shows three distinct peaks with the first well below 4Å indicating compression of the electronic wavefunctions by pressure. The remaining densities all show a first peak around 4Å, broadening with pressure but with only weak radius dependence. We take this to be the van der Waals diameter of Kr, consistent with textbook values of 4.04Å. The position of the second peak shows strong distance dependence and is barely discernible above 100Å/atom.

The van der Waals correction has a significant effect in increasing the height of the first peak, whereas longer range structure appears similar. This is unsurprising as the vdW correction is implemented as a short-ranged interaction between pairs of molecules.

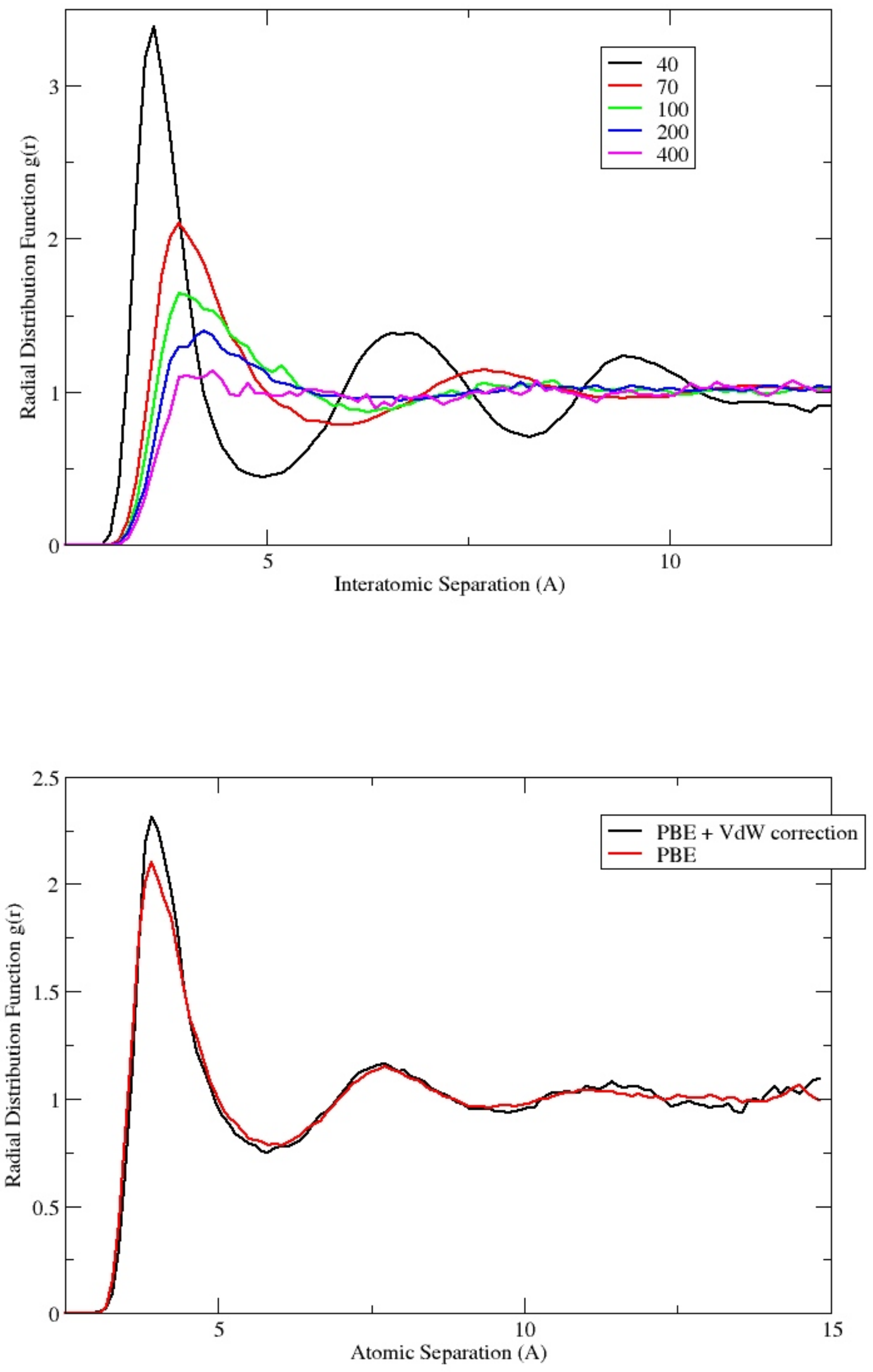

Figure 22: (Top) Pair Distribution Functions from density functional theory calculation. Legend indicates densities volume per atom 40, 70, 100, 200, and 400 Å^3. (Bottom) Pair Distribution Functions from density functional theory with and without van der Waals corrections for a density of 70 Å/atom.

References

(1) Teitsma, A.; Egelstaff, P. Three-body-potential Contribution to the Structure of Krypton Gas. *Physical Review A* **1980**, *21*, 367.
